# Oxidation State Dependence of the Actinide M_4/5_-Edge XANES of Actinyl (An = U, Np, Pu) Systems

**DOI:** 10.1021/acs.inorgchem.5c05776

**Published:** 2026-04-01

**Authors:** Kurtis Stanistreet-Welsh, Andrew Kerridge

**Affiliations:** Department of Chemistry, 4396 Lancaster University, Lancaster LA1 4YB, U.K.

## Abstract

Restricted Active Space simulations including spin–orbit coupling are used to predict the An M_4/5_-edge XANES spectra of uranyl, neptunyl and plutonyl ions in the +5 and +6 oxidation states. The simulations reproduce the characteristic red-shift in XANES upon lowering the actinide oxidation state. This shift arises from relative changes in the ground- and core-excited state (CES) energies between An­(VI) and An­(V), which can be interpreted in terms of electron affinities. QTAIM analysis links the electron affinity of An­(VI) states to actinide electron localization and to the more familiar concept of effective nuclear charge. The lower excitation energy in An­(V) species stem from a comparatively high effective nuclear charge in An­(VI) CESs and lower charge in An­(V) ground states, producing enhanced electron affinity in the former and reduced core–electron binding in the latter. These combined effects yield more accessible CESs in An­(V), manifesting as a red-shift. This study also examines the proposed correlation between ground-state covalency and the separation between 5f-δ/ϕ and 5f-σ* peaks. Although experimental trends suggest decreasing covalency across the series, QTAIM metrics do not support this. Instead, reduced covalency correlates with smaller peak separations only when the oxidation state is lowered within a given actinyl system.

## Graphical abstract

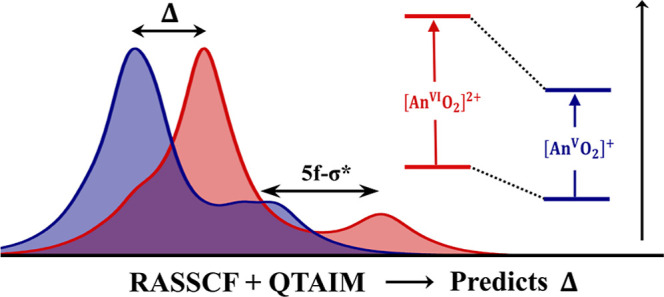

## Introduction

Oxidation state is a key property in understanding both the physical and chemical properties of actinide species.^1–7^ For instance, actinide oxidation state is a determining factor in the design of new materials used for long-term nuclear waste storage,^8–16^ in the separation of spent nuclear fuels,^17–20^ and in the development of remediation and immobilization strategies for contaminated sites.^21–31^ The early actinyls are one of the prototypical models for understanding actinide covalency and can exist in a number of oxidation states, including the high +6 and +5 states.^3,4,32–45^ Determining the behavior of actinyls in various oxidation states becomes crucial in the event that nuclear material or waste breaches controlled confinement. In these scenarios, actinyl ions can form and easily migrate through the natural environment spreading contamination.^2,46–48^ Immobilisation strategies can be used to target the reduction of actinides to lower oxidation states which exhibit limited solubility, inhibiting migration from contaminated sites.^13,14,21–23,25,27–30,49–51^

X-ray absorption near-edge structure (XANES) has emerged as a highly capable spectroscopic tool for probing actinide electronic structure and, in particular, An M_4/5_-edge XANES has become well-established for determining actinide oxidation state for a number of species with both high sensitivity and spectral resolution.^10,25,27,37,49,52–64^ In An M_4/5_-edge XANES, peak intensity is driven by excitation of core electrons from actinide 3d-orbitals into actinide 5f-based valence orbitals. The energy difference between these core 3d and partially empty 5f-valence orbitals changes upon the addition or removal of electrons when altering the actinide oxidation state and manifests as shifts in the position of all XANES peaks. These characteristic shifts in XANES spectra can be utilized to determine the actinide oxidation state by comparing the resulting spectrum with those of known reference compounds.^49^ In actinide systems exhibiting strong covalency, hybridization of actinide 5f-orbitals with those of the ligands generates bonding and antibonding molecular orbitals which are also accessible via core-excitation, leading to pre-edge features in XANES spectra.^49,52,65^ These features are useful for probing the nature of actinide-ligand bonding, containing information about relative orbital energies as well as the magnitude of orbital hybridization. The pairing of An M_4/5_-edge XANES with theoretical simulations has proven highly successful in characterizing pre-edge features and providing key insights into the nature of actinide-ligand bonding,^66^ with particular interest in the literature on the actinyls and actinide oxides.^27,37,49,50,52,58,60,67–80^ To date, a number of studies have clearly demonstrated the occurrence of energy shifts in the An M_4/5_-edge spectra of actinyls due to a change in oxidation state.^49,50,58,60,80,81^ The energy shift is typically rationalized as being due to changes in the actinide effective nuclear charge upon the addition or removal of electrons, which alters the binding energy of the 3d core–electrons and therefore the energy required to induce an excitation.^50,60,71,82^ Impacts on actinyl covalency due to a change in oxidation state have also been explored using An M_4/5_-edge XANES, with studies indicating a potential correlation between axial covalency and the relative separation between peaks assigned to the nonbonding 5f-δ/ϕ orbitals and the 5f-σ* peak.^50,52,58,60,69^

Given the rich electronic structure information contained within experimental XANES spectra, there is a need for robust and accurate simulation techniques to aid in meaningful interpretation of the resulting spectra. In recent years, multiconfigurational Restricted Active Space Self-Consistent Field^83–85^ (RASSCF) simulations have been utilized to successfully simulate the XANES spectra of a number of actinide systems.^86–93^ Full relativistic multiconfigurational treatments of XANES simulations have also been performed for actinide systems and have similarly proven insightful, and in-particular, emphasize the need to capture the multiconfigurational nature of the CESs.^94–97^ Previous studies, including our own work, have simulated An M_4/5_-edge XANES of the actinyls, providing insight into the nature of the states being probed within the context of actinide-ligand bonding.^86,90,91,93^ While these studies have demonstrated the ability of RASSCF to accurately predict the XANES spectrum of actinide systems, to date, no RASSCF-based studies have demonstrated the key energy shifts in spectra due to changes in actinide oxidation state. More recently,^98^ Kubin et al. have demonstrated the ability of RASSCF simulations to correctly predict the variation of L-edge XANES due to a change in oxidation state in Manganese (Mn) transition metal complexes. In the present contribution, we demonstrate the ability of RASSCF simulations to correctly predict the variation in An M_4/5_-edge XANES spectra of actinyls resulting from a lowering of oxidation state from An­(VI) to An­(V). Kubin proposed that shifts in XANES spectra are primarily attributed to differences in electron affinity between the GS and CESs, with relative energies rationalized in terms of factors such as the differences in charge and spin densities, Coulomb interactions, and orbital contractions. In the present study, we demonstrate a clear connection between the relative energies of key states measured by XANES to determine oxidation state and the metrics obtained from Bader’s Quantum Theory of Atoms in Molecules^99,100^ (QTAIM), which can be directly interpreted in the more familiar context of changes in effective nuclear charge. Finally, the impact on actinyl covalency due to a change in oxidation state and the manifestation of these changes as features in the simulated XANES spectra are examined.

## Computational Details

Scalar relativistic multiconfigurational An M_4/5_-edge XANES simulations were performed using version 21.02 of Openmolcas^101,102^ on [AnO_2_]^2+/+^ (An = U, Np, Pu) systems using the all-electron relativistic ANO-RCC TZVP basis sets of Roos et al.^103,104^ Higher angular momentum h-functions were removed from actinide centers to enable compatibility with analysis software. Multiconfigurational simulations made use of the second order Douglas-Kroll-Hess Hamiltonian to account for scalar relativistic effects and Cholesky decomposition was utilized throughout to speed-up integral calculations.^105–108^ Restricted Active Space Self-Consistent Field (RASSCF) theory^83–85^ calculations were utilized to obtain the states required for XANES simulations. The active space was constructed by placing the even (g)-parity 3d core-orbitals in RAS1, the odd (u)-parity valence bonding orbitals in RAS2, and both the nonbonding 5f and antibonding valence orbitals of u-parity in RAS3, as shown in Figure 1. Built-in supersymmetry designations of Openmolcas were utilized to ensure core-orbitals remained within RAS1 during the SCF procedure. The actinyl­(VI) ground state 5f orbital occupations (*n*) originate from those of the An^6+^ ion, with *n* = 0, 1, and 2 for U, Np, and Pu, respectively. Like-wise, the actinyl­(V) ground states (An^5+^) have *n* = 1, 2, and 3 for U, Np, and Pu, respectively. In Sauri notation,^109^ RAS­(SD) grounds state (GS) calculations correspond to RAS­(16 + *n*, 0, 2 + *n*; 5, 3, 7) and core excited state (CES) calculations correspond to RAS­(16 + *n*, 1, 2 + *n*; 5, 3, 7). To obtain the GS and CESs of various spin-multiplicities and state-symmetries, state-averaged (SA) RASSCF calculations were performed followed by state-specific second order RAS perturbation theory (SS-RASPT2) with a default IPEA shift of 0.25 au and imaginary level shift of 0.5 au in order to recover dynamical correlation and generate quantitative state energies.^109–112^ The chosen shift values offered a reasonable balance between converging intruder free solutions without introducing significant shift bias to RASPT2 energies and are comparable to those used in related studies.^86,87,89–91,93^ The RASSI formalism was utilized to generate Spin–Orbit (SO) coupled states via state-interaction of the scalar relativistic spin-free GS and CESs with a mean-field SO operator using atomic mean-field integrals (AMFI) within the RASSI formalism.^113,114^ RASPT2 state energies were provided to RASSI calculations for use in Hamiltonian matrix formulation. RASSI calculations provide spin–orbit coupled state energies as well as corresponding oscillator strengths between the GS and CESs, utilized to plot transition stick spectra which are subsequently broadened by fitting Lorentzian functions with a full-width at half-maximum (fwhm) of 1.5 eV to each transition stick. An assessment of alternative broadening schemes, including Gaussian and Voigt profiles over a range of fwhm values, is provided in the Supporting Information. The energy position of the peak maxima are found to be largely invariant with respect to both the chosen fwhm and the specific broadening scheme employed. The selected Lorentzian fwhm of 1.5 eV is consistent with the value previously employed by Polly et al.^93^ for similar RAS-based simulations of uranyl and provides representative spectral profiles comparable with available high-resolution XANES experiments,^27,50,52,57,58,60^ where the effective spectral resolution is no longer strictly limited to the intrinsic core-hole lifetime. Spin orbit natural orbitals^86,115,116^ (SONOs) of SO states were utilized for peak assignment and for additional QTAIM analysis, performed using version 19.02.13 of AIMALL.^117^

**Figure 1:**
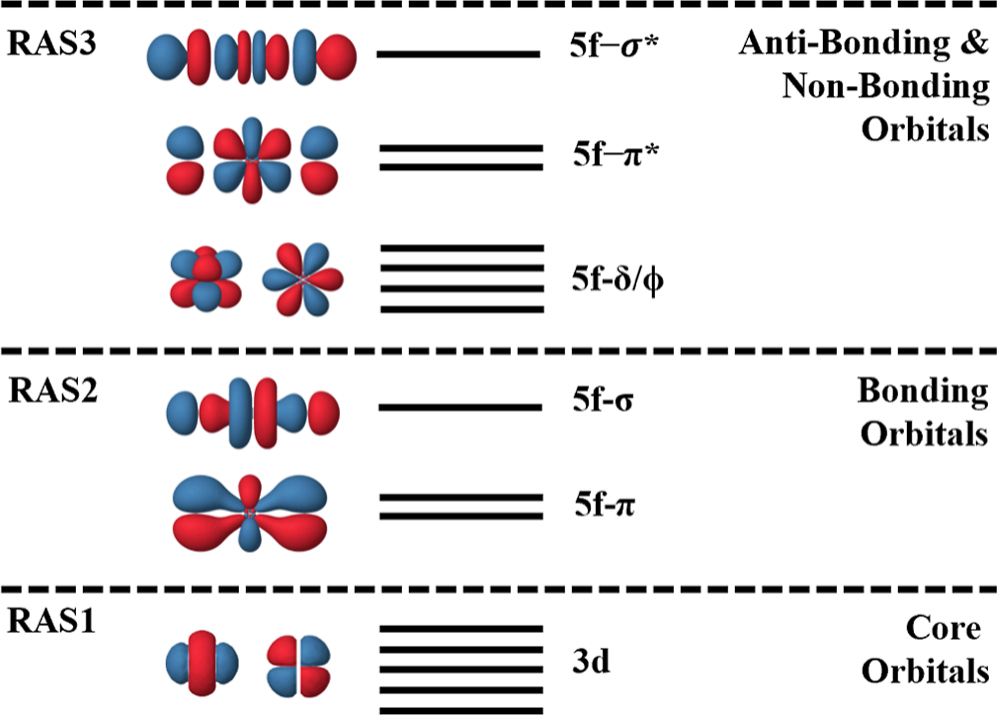
Schematic of pseudonatural orbitals included within the active space setup for RASSCF XANES simulations. Qualitative levels indicate the number of orbitals assigned to an orbital label and do not reflect actual energy positions.

Bare actinyl [AnO_2_]^2+/+^ systems were constructed with bond lengths set to those of PBE0/def-TZVP optimized actinyl penta-aquo complexes performed using version 6.6 of TURBOMOLE with a continuum solvent model used to account for solvation effects.^118–123^ For additional computational details, including active space considerations and constraints, DFT optimizations, state-averaging in SA-RASSCF calculations and RAS­(S) simulation results, see the Supporting Information. No uncommon hazards are noted.

## Results and Discussion

### Actinyl Structures and Ground-State Characterization

In this study, the actinyl penta-aquo complexes were chosen as the experimentally relevant structures from which to define bond lengths of the bare actinyl models used in XANES simulations. The choice of actinyl penta-aquo complexes was motivated by the experimental work of Vitova et al., who reported a set of An M_4/5_-edge XANES spectra across the actinyl­(VI) series from uranyl to plutonyl.^52^ These experiments were performed in aqueous solution where actinyls typically form penta-aquo dication complexes whereby up to five water molecules comprise the primary solvation shell via equatorial binding to the actinyl dications. Theoretical actinyl An-O axial bond lengths of 1.74, 1.72, and 1.70 Å were obtained for uranyl­(VI), neptunyl­(VI) and plutonyl­(VI), respectively. Similarly, bond lengths of 1.81, 1.79, and 1.77 Å were obtained for uranyl­(V), neptunyl­(V) and plutonyl­(V) systems, respectively. In both An­(VI) and An­(V) actinyls, there is a consistent 0.02 Å change in bond length moving across the actinide series. This variation is reasonable when compared to the 0.01 Å change in actinly­(VI) bond lengths for aquo-complexes from uranyl to plutonyl reported by others.^2,52^ Similarly, the differences in theoretical bond lengths between actinyl­(VI) and actinyl­(V) systems is 0.07 Å, and reflects the 0.07–0.08 Å change found in neptunyl and plutonyl aquo-complexes in other studies.^124–126^

Based on the occupancy of spin–orbit natural orbitals (SONOs) for the multiconfigurational spin–orbit GSs, [UO_2_]^2+^ can be characterized in terms of the nonbonding 5f orbital occupancy (5f-δ/ϕ) as a singlet (5f-δ)^0^(5f-ϕ)^0^ state. The [NpO_2_]^2+^ and [PuO_2_]^2+^ GS electronic configurations, isoelectric to [UO_2_]^+^ and [NpO_2_]^+^, are characterized as (5f-δ)^0^(5f-ϕ)^1^ and (5f-δ)^1^(5f-ϕ),^1^ respectively. [PuO_2_]^+^ corresponds to a (5f-δ)^2^(5f-ϕ)^1^ electronic configuration. The electronic configurations, combined with evaluation of Ω quantum numbers, enables assignment of molecular term symbols to the spin–orbit coupled GSs. [UO_2_]^2+^ is assigned to a singlet ^1^Σ_0g_^+^ state. Both [NpO_2_]^2+^ and [UO_2_]^+^ give degenerate doublets assigned as ^2^Φ_5/2u_. The [PuO_2_]^2+^ and [NpO_2_]^+^ GSs are assigned as triplet ^3^H_4g_ molecular states, and the [PuO_2_]^+^ GS is assigned to degenerate quartet ^4^Φ_3/2u_ states. These GS characterisations are in agreement with those reported by others.^2,33,94,127^

### Simulated An M_4/5_-Edge XANES

Figure 2 presents the RAS­(SD) simulated XANES spectra for the actinyls in the +6 and +5 oxidation states. Simulated spectral profiles for actinyl­(VI) spectra are characteristic of both experimental U M_4_-edge and Np/Pu M_5_-edge XANES reported by Vitova et al.,^52^ but also of simulated spectra reported by Autschbach and co-workers.^86^ Profiles for actinyl­(V) spectra cannot be directly compared with experimental profiles for actinyl aquo-complexes since to the authors best knowledge none-to-date have been reported, but simulated profiles are in good general agreement with those of other actinyl containing compounds.^27,50,52,57,58,60^ As we have demonstrated previously,^90,91^ peak assignments can be performed by examining the natural occupations of SONOs for CESs associated with the most intense transitions. The uranyl­(VI) M_4_-edge XANES spectrum consists of three resolved peaks. The lowest energy peak is assigned to core-excitations into the 5f-δ/ϕ nonbonding orbitals, the next into the 5f-π* orbitals, and the highest into the 5f-σ* orbital. All other spectra in Figure 2 contain a main peak and a second resolved peak. The higher energy peak can, in all cases, be attributed to core-excitations into the 5f-σ* orbital. The lower energy and most intense feature arises from transitions involving core-excited states with both 5f-δ/ϕ and 5f-π* occupancy. Therefore, the peak assignment in Figure 2 reflects this.

**Figure 2:**
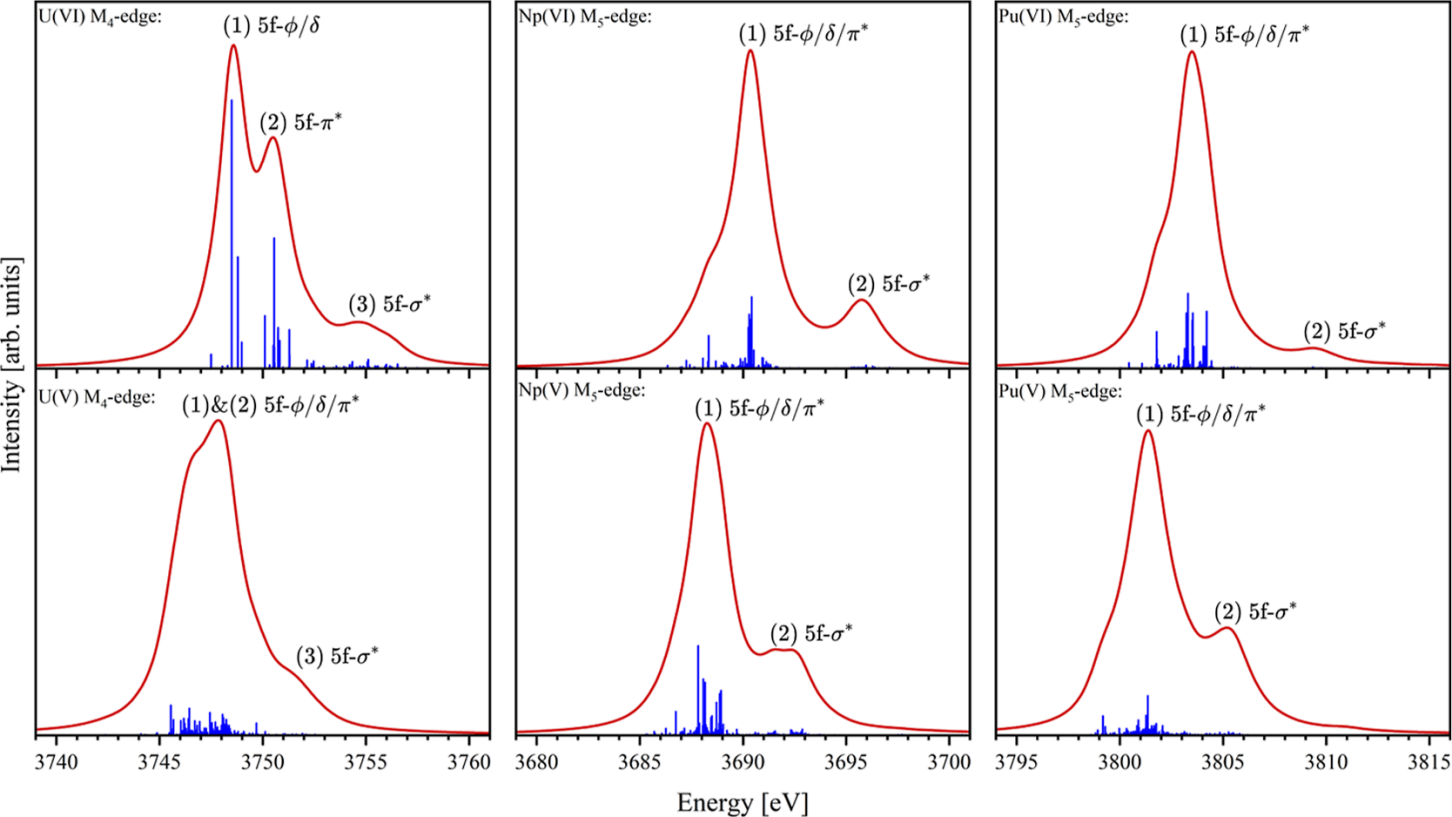
Simulated An M_4/5_-edge XANES spectra for actinyls in the +6 and +5 oxidation states. Plots include transition sticks (blue) which indicate the excitation energy and oscillator strength of core-excitations and the XANES spectrum (red) generated by Lorentzian broadening of transitions. From left to right, plots show uranyl M_4_-edge, neptunyl and plutonyl M_5_-edge XANES in the +6 (top) and +5 (bottom) actinide oxidation states.

RAS­(S) spectra and assignments presented within Figure S22 of the Supporting Information confirm a similar characterization of simulated peaks. Across all the simulated XANES spectra, the 5f-σ* peak is generated from a large collection of very low intensity transitions that combine upon Lorentzian broadening to give a distinct peak feature. This appears to be a feature of the RAS­(SD) simulation setup since clear high intensity transitions are found at the RAS­(S) level but not at RAS­(SD). The true scale of the transitions for 5f-σ* peaks is best viewed in Figures S8–S16 of the SI for RAS­(SD) simulations. Overall, the relative peak intensities are found to follow the degree of 5f-character in the acceptor orbitals, with peaks associated with the localized 5f-δ/ϕ orbitals having the greatest intensity. For example, the 5f-π* peak resolved in the uranyl­(VI) U M_4_-edge spectrum has a lower relative intensity than the 5f-δ/ϕ peak, due to a degree of oxygen 2p-orbital mixing which reduces the 5f-character. The 5f-σ* peaks are of lowest intensity since the acceptor orbitals tend to have the smallest 5f-character, with close to 50:50 metal/ligand orbital mixing, as demonstrated in previous works.^90,91^ The overall peak assignments for the simulated actinyl­(VI) spectra are in agreement with those reported for experimental spectra of actinyl­(VI) aquo-complexes,^52^ while simulated actinyl­(V) spectra are found to be in good agreement with assignments of experimental spectra performed on a variety of different actinyl­(V) containing compounds.^27,50,52,57,58,60^ A change in oxidation state was not expected to alter peak assignments since the antibonding orbital ordering remains unchanged, and this is the case across the simulated spectra. The CESs generated by RAS­(SD) simulations present significant multiconfigurational character, which manifests as redistribution of electrons from bonding orbitals into the nonbonding 5f and antibonding orbitals to varying degrees. This multiconfigurational character becomes more pronounced both in higher energy CESs and as the number of electrons occupying the nonbonding 5f orbitals increases. Detailed assignment can be found in Tables S9 and S10 of the Suppoting Information.

### Predicting An M_4/5_-Edge XANES Shift Behavior

The most substantive change in XANES spectral profile due to a change in actinide oxidation state occurs for the U M_4_-edge spectra, with three well-resolved peaks for uranyl­(VI) reducing to two in the uranyl­(V) species, where the peaks attributed to 5f-δ/ϕ and 5f-π* transitions of the former merge into a single peak in the latter. Figure 3 presents the XANES spectra for each actinyl system in the +6 and +5 oxidation state to demonstrate the ability of simulation to predict the expected shift behavior of XANES spectra due to a change in actinide oxidation state. Reduction of a system to lower oxidation state results in a red-shift of the resulting spectrum. This shift is well-documented and is the basis for oxidation state determination using XANES.^49,50,58,60^ Figure 3 demonstrates that the RAS­(SD) simulations capture the correct red-shift behavior for all three actinyl systems upon reduction from +6 to +5. A shift-value (Δ) of approximately 2 eV across the three systems is reported and was calculated by measuring the energy difference between the positions of the first peak in each of the spectra. Although a shift value of ∼2 eV is not unreasonable for a change in oxidation state,^49^ shifts from a range of experimentally relevant references for An­(VI/V) couples report shifts in the range of 0.4–0.6 eV,^53,55,58,60^ suggesting a potential ∼1 eV overestimation in the simulations. Bond length differences between the An­(VI) and An­(V) species in experimental studies are on the order of ∼0.1 Å,^53,55,58,60^ which is comparable to the 0.07 Å change in the DFT derived structures. This minor discrepancy in theoretical and experimentally relevant An–O bond lengths is therefore thought to have negligible impact on the shift overestimation. Instead, the difference is suspected to originate from neglecting equatorial ligands and other aspects of the chemical environment in which the XANES measurements are performed. Previous studies have indicated that the key spectral features of actinyl XANES are sufficiently predicted using actinyl ions in the gas phase, but the relative energy position of peaks are found to differ when accounting for other coordinating ligands.^86,90,91,97,128,129^ For instance, a M_5_-edge simulation using comparable RAS methods for a plutonyl aquo complex by Autschbach and co-workers yielded a ∼10 eV improvement in the absolute energy position of the XANES spectrum with respect to the experiment when performed in aqueous conditions compared with simulations using gas-phase actinyl ions.^86^ Despite the potential overestimation of Δ in comparison to particular experimental studies, the key red-shift behavior is successfully captured by the current simulations and affords the ability to investigate the origin of the shift by probing both the GSs and CESs involved.

**Figure 3:**
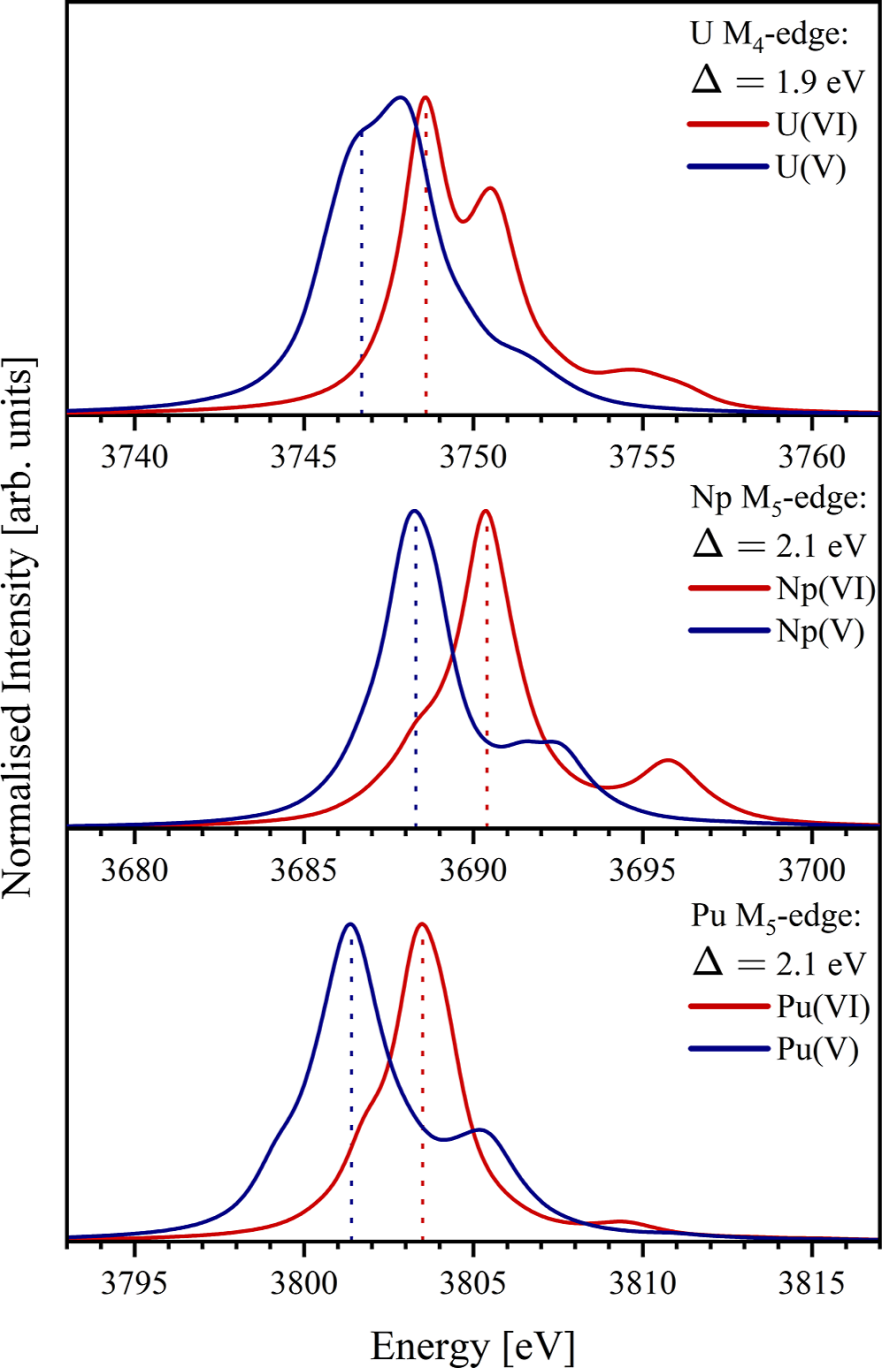
Simulated U­(VI/V) M_4_-edge, Np­(VI/V) and Pu­(VI/V) M_5_-edge XANES spectra, rendered to illustrate the predicted shift behavior (Δ) of spectra due to a change in actinide oxidation state. Intensity is normalized for ease of comparing energy shifts. Vertical dashed lines indicate the energies used to calculate the shift value Δ.

### A Mechanism for XANES Oxidation Shifts

A shift in the peak positions of a XANES spectrum due to a change in the oxidation state of the absorbing center is typically rationalized in terms of electrostatic considerations. Literature consensus attributes the shift in XANES spectra to a change in core–electron binding energies due to variations in the effective nuclear charge and electron shielding of core–electrons for the absorbing atom upon a change in oxidation state.^50,60,71,82^ For instance, upon oxidation the removal of an electron increases the effective nuclear charge of the absorbing center, drawing the core–electrons closer to the nucleus and thereby increasing their binding energy, such that the energy required for core excitation rises and the resulting spectral features are blue-shifted.

A more nuanced explanation for the observed XANES shifts has been proposed by Kubin et al. in the context of Manganese (Mn) L-edge XANES, which probes 2p → 3d transitions.^98^ Here, the shift is attributed among other factors to differences in electron affinity between key states assigned to the most intense peaks in the Mn­(III) and Mn­(II) L-edge spectra. By examining the GSs and CESs of these systems, a greater electron affinity among the CESs in comparison to the GSs resulted in a more energetically accessible CES in the reduced species, and hence a lower excitation energy. Kubin rationalized the relative state energies by considering differences in charge and spin densities, which pointed to a compression of the valence 3d orbitals, and by considering overall changes to Coulomb and Exchange interactions. However, our analysis deviates from that of Kubin in favor of utilizing QTAIM metrics for the actinide center in both the ground- and core-excited states to rationalize the relative state energies and, by extension, the binding energy of the core electrons due to actinide effective nuclear charge.

Figure 4 plots the total energy for the ground- and core-excited states associated with the first peak in the An M_4/5_-edge XANES spectra for the actinyls in the +6 and +5 oxidation states. The relative energy positions of the An­(VI) and An­(V) ground- and core-excited states are similar to those Kubin identified for the Mn­(III) and Mn­(II) systems, suggesting the electron affinity of the actinide centers can also explain the actinyl XANES shifts. For actinyl GSs, the addition of an electron upon reduction from An­(VI) to An­(V) (i.e., from a 3d^10^5f^*n*^ to 3d^10^5f^*n*+1^ configuration), results in a relatively more stable An­(V) state compared with An­(VI), as shown in Figure 4. Similarly, An­(V) CESs are relatively more stable than those of the An­(VI) CESs. The energy difference between these states, denoted as E_EA_ in Figure 4, represents the (adiabatic) electron affinity of the An­(VI) GS or the An­(VI) CES (depending on the state of interest). For every actinide system considered, there is a greater energetic difference between the CESs than between the GSs, or like-wise a greater electron affinity in An­(VI) CESs than in An­(VI) GSs. The overall result is a smaller energy gap (excitation energy) between the GS and CES in An­(V) species. This is demonstrated clearly in the Figure 4, with the energy difference between the An­(VI) and An­(V) GSs in the range of 15–17 eV, while the energy difference between the CESs is between 17 and 19 eV, giving an approximate 2 eV stabilization of the CES relative to the GS in the An­(V) species. This 2 eV difference manifests as a lower excitation energy in the actinyl­(V) system and is approximately equal in value to the predicted peak shifts (Δ) identified in the simulated spectra (Figure 3). Note that the small discrepancy in shift values reported by these two approaches is due to the simulated peaks in Figure 3 being a result of the broadening of a large number of transitions rather than single transitions associated with the states presented in Figure 4.

**Figure 4:**
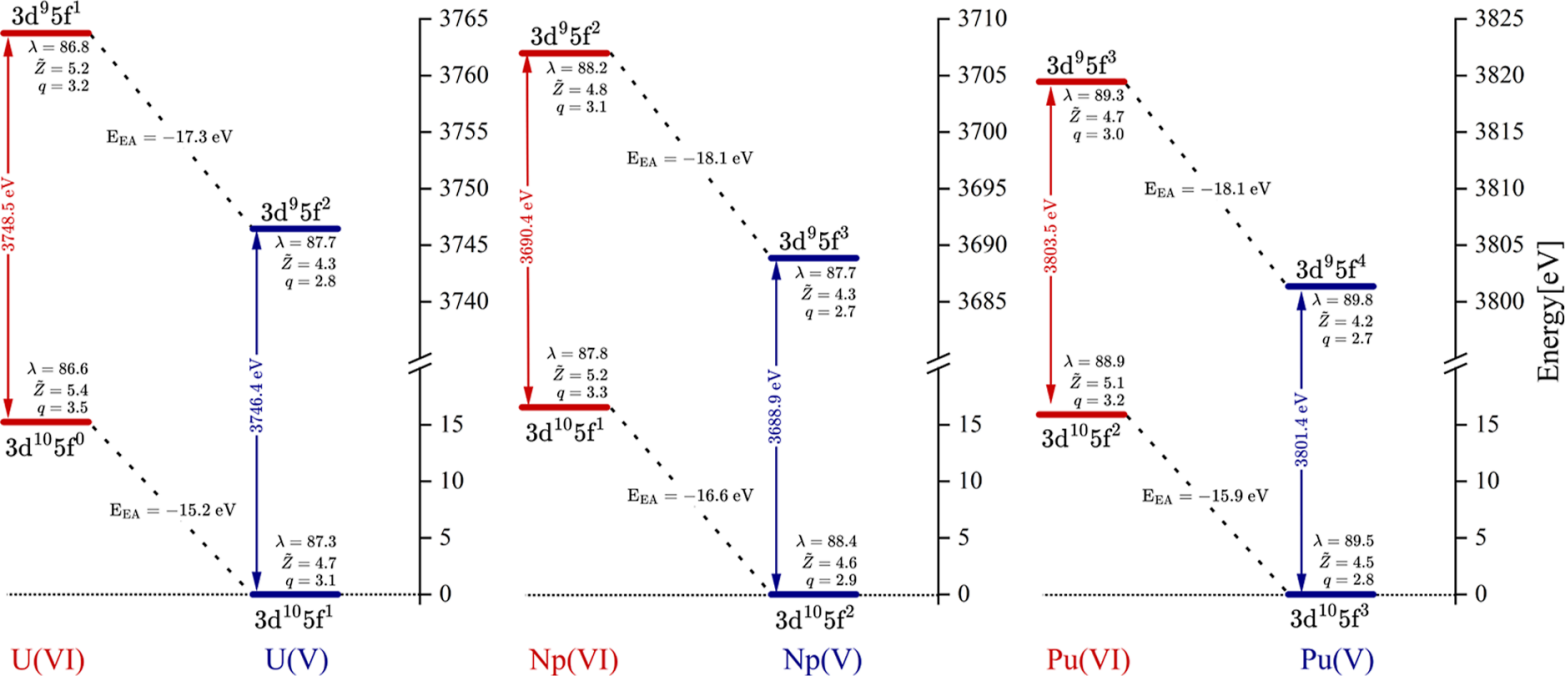
Total state energy level diagrams plotting spin–orbit coupled states obtained from RASSI calculations for An M_4/5_-edge XANES simulations of actinyls in the +6 and +5 oxidation states. Each diagram from left to right plots the relative state energy of the spin–orbit coupled ground state and the core-excited state for the most intense transition associated with the first peak in the uranyl­(VI/V) M_4_-edge, neptunyl­(VI/V) M_5_-edge, and plutonyl­(VI/V) M_5_-edge XANES spectra, respectively. For each state, the diagrams include the localization index λ, atomic charge *q*, and a QTAIM derived quantity reflecting effective charge Z̃ for the actinide center. The E_EA_ values represent the electron affinity. Energies are given in eV and charges in a.u.

Figure 4 also gives the localization index λ_An_ and the QTAIM atomic charge *q*_An_ for the actinide (An) centers in the different ground- and core-excited states.^99,100^ λ quantifies the number of electrons localized on an atomic center, as defined by its QTAIM atomic basin. A parameter ${\mathrm{Z̃}}_{An}$ is also given and defined as

1 $${\mathrm{Z̃}}_{An}=Z_{An}-\lambda_{An}$$

where *Z*_An_ is the atomic number of the actinide, with values of 92 to 94 from U to Pu. The quantity ${\mathrm{Z̃}}_{An}$ has been previously shown by our group to correlate with changes in oxidation state.^45,130–132^ In the present work, we employ the dimensionless quantity ${\mathrm{Z̃}}_{An}$ to represent the different effective nuclear charges experienced at a given actinide center if λ_An_ is utilized as a means by which to quantify variation in the degree of electron shielding between different states.

QTAIM analysis performed on the states highlighted in Figure 4 finds the U­(VI) 3d^10^5f^0^ GS to have a lower λ_An_ value of 86.6 compared to a value of 87.3 for the U­(V) 3d^10^5f^1^ GS. In the context of eq 1, this represents a decreased electron shielding and therefore a greater effective nuclear charge (as measured by ${\mathrm{Z̃}}_{An}$ ) on the U­(VI) center compared with U­(V). This is also reflected in the actinide atomic charge which is greater for U­(VI) compared with U­(V). Conceptually, a greater effective nuclear charge will lead to an enhanced electron affinity since a more positively charged center has a greater potential to attract and bind an electron. Therefore, the stabilized GS in U­(V) relative to U­(VI) in Figure 4, reflects the affinity of the U­(VI) species to bind an additional electron due to its greater effective nuclear charge (as quantified by the differences in ${\mathrm{Z̃}}_{An}$ ). The same applies to the GSs of the Np and Pu species, with the higher oxidation state species having the greater ${\mathrm{Z̃}}_{An}$ value, therefore an enhanced electron affinity, thereby leading to stablised An­(V) states. This argument also rationalizes the relative differences in CES energies in the actinyls of different oxidation states, with the ${\mathrm{Z̃}}_{An}$ values found to be greater in the An­(VI) CESs compared to the An­(V) CESs.

The different excitation energies required to access the CESs from the GS in the actinyl­(VI) and actinyl­(V) systems, and thus the XANES shift, can also be rationalized by considering the electron localization (λ_An_) on the actinide centers. The excitation energy required to generate a CES of the form 3d^9^ 5f^*n*+1^ from a GS of the form 3d^10^ 5f^*n*^ for a given actinyl system is dependent on the binding energy of the 3d core–electrons in the GS. The λ_An_ values indicate a smaller electron localization in the An­(VI) GS compared to An­(V), indicating a greater effective nuclear charge (confirmed by larger ${\mathrm{Z̃}}_{An}$ values) in the former and therefore a greater binding energy. The greater binding energy in the An­(VI) GS requires a larger excitation energy to form the CES, and accounts for the shift in XANES peaks to higher energy relative to An­(V) actinyls. These differing core binding energies, rationalized through QTAIM analysis, also explain in-part the greater values of E_EA_ (17–19 eV) reported in Figure 4 for the CESs as compared with the GSs (15–17 eV), since if the relative energy difference (excitation energy) is larger between the An­(VI) GS and CES, then by necessity the An­(V) CES must be relatively stabilized with respect to its GS for this energy relationship to hold. This indirectly accounts for the differing magnitudes of the electron affinities and indicates the core–electron binding in the GSs to be a contributing factor to these differences. This is also important to note, since if the ${\mathrm{Z̃}}_{An}$ values are examined only in the GS and CES of a particular An­(VI) system, in for example uranyl, the ${\mathrm{Z̃}}_{An}$ value in the GS (+5.4) is greater than that of the CES (+5.2), and one might expect an enhanced electron affinity in the former in line with the effective nuclear charge arguments discussed. However, the opposite is the case. Therefore, only considering the different ${\mathrm{Z̃}}_{An}$ values of the GS and CES in a particular oxidation state appears to be insufficient to explain the greater affinity of the CES to bind an additional electron compared with the GS. A more direct rationalization would likely require an investigation into additional factors affecting electron affinity, such as the contraction of 3d- or the availability of 5f-based orbitals in the GS or CES, for example.

The shift in XANES spectra between two species can also be affected by variations in bonding interactions.^49^ In the absence of ligand interactions, a change in oxidation state from An­(VI) to An­(V) might be expected to be reflected in a unit change in the actinide localization index λ_An_.^130^ As discussed, the red shift in XANES spectra due to a change in oxidation state originates from the relative differences in energy between the GS and CESs associated with the An­(VI) and An­(V) species as shown in Figure 4, and rationalized by variations in effective nuclear charge as defined by ${\mathrm{Z̃}}_{An}$ in eq 1. Deviations from a unit change in λ_An_ can therefore be examined to infer the degree to which interactions with oxygen ligands may play a role in the red-shift of XANES spectra due to a change in oxidation state.

To measure changes in An-O bonding between An­(VI) and An­(V) systems, the electron density at the bond critical point ρ_BCP_ and the delocalization index δ­(An,O), a measure of the number of electrons shared between actinide and oxygen atomic basins, can be utilized. We have previously applied these QTAIM metrics to quantify changes in F-element bonding and electron localization in both ground- and excited-states.^132,133^ This analysis was also applied more recently to analyze actinyl core-excited states in the context of XANES simulations.^90,91^

QTAIM analysis performed on the actinyl GSs indicates that a change in oxidation state from An­(VI) to An­(V) results in an increase in λ_An_ and λ_O_ of approximately 0.65 and 0.39, respectively, while the δ­(An,O) values are decreased by approximately 0.20. Note that in order for the total change in electron count between An­(VI) and An­(V) to sum to one, the λ_O_ and δ­(An,O) values must be multiplied by a factor of 2, reflecting the presence of two O centers, giving changes of ∼0.78 and ∼0.40, respectively. This is reported in Table 1. A full account of QTAIM analysis is given in the SI and similar QTAIM metrics are found for the core-excited states associated with the peaks used in measuring the XANES shifts in Figure 3.

**Table 1:** Change in Delocalisation Δδ­(An,O)­and Localisation Index for the Actinide Δλ_An_ and Oxygen Δλ_O_ Centers in the Actinyls Upon Reduction from the +6 to +5 Oxidation State: An­(VI) → An­(V)^a^

An(VI) → An(V)	Δλ_An_	2 × Δλ_O_	2 × Δδ(An,O)
U(VI) → U(V)	+0.66	+0.78	–0.42
Np(VI) → Np(V)	+0.65	+0.82	–0.44
Pu(VI) → Pu(V)	+0.64	+0.82	–0.44

a Values are obtained from the spin–orbit coupled ground states.

Considering both λ and δ metrics together in Table 1 indicates that the addition of an electron to actinyl­(VI) systems does not lead to a unit increase in λ_An_ for the An­(V) center. Instead, the additional electron leads to increased localization across the three atomic centers in actinyl­(V) systems. At the same time, values of δ­(An,O) are decreased for actinyl­(V) indicating reduced electron sharing. The electron density at the An–O bond critical point, ρ_BCP_(An,O), also decreases upon actinide reduction (Table 2). This finding indicates that the less than unit change in λ_An_ (∼0.65) is not a consequence of enhanced covalent bonding with oxygen ligands via actinide-to-ligand electron donation. Instead, the reported Δλ_An_ value is simply a consequence of the oxygen ligands playing a role in the optimal distribution of charge across the three-center system. Therefore, future work aiming to predict the energy shifts in XANES due to a change in oxidation state for specific actinyl complexes may consider the inclusion of equatorial ligands to better facilitate charge redistribution upon reduction/oxidation to reflect changes in the real systems. The expected result of which would be a better reflection of the actinide effective nuclear charges in the real systems, thereby leading to better relative state energies, and therefore relative shifts in XANES spectra which closer match experimental measurements.

**Table 2:** Electron Density at the Bond Critical Point, ρ_BCP_(An,O), and Delocalisation Index, δ­(An,O), for An-O Bonds in the Spin–Orbit Coupled Ground State^a^

	ρ_BCP_(An,O)		δ(An,O)	
system	An(VI)	An(V)	An(VI)	An(V)
U	0.35	0.29	1.86	1.65
Np	0.37	0.30	1.89	1.67
Pu	0.38	0.31	1.90	1.68

a Values of ρ_BCP_ are in atomic units.

### Implications for Actinyl Covalency

A number of studies have proposed a correlation between actinyl axial covalency and the relative energy separation of the 5f-σ* and 5f-δ/ϕ peaks (herein referred to as the 5f-σ* peak separation).^50,52,54,58,60,69,134,135^ A larger peak separation is interpreted as being due to a greater destabilization of the 5f-σ* orbital via enhanced effects of the “pushing from below” (PFB) mechanism, and indicates a strengthening of 5f-σ covalency.^41,50,52,80,136^ While there is a number of studies which support a potential correlation, others note that this correlation may not hold in all cases, and therefore a complete consensus has yet to be formed.^79,129^ Vitova et al.^52^ has investigated the 5f-σ* peak separation as an indicator of axial covalency across the actinyl­(VI) series from U to Pu. Peak separation was shown to decrease across the series, interpreted in the context of the PFB mechanism as a decreasing axial bond covalency. However, this finding appeared to contradict studies that point to an increasing covalency across the series. Autschbach and co-workers^86^ partially resolved this puzzle by demonstrating that the peak separation across the series better reflects a decreasing covalent orbital mixing character for the 5f-σ bonding orbital in the CES as opposed to the GS. In this section, we investigate whether a relationship between the 5f-σ* peak separation and actinyl covalency can be established using QTAIM analysis.

Figure 5 plots the energetic separation between the 5f-σ* and 5f-δ/ϕ peaks (5f-σ* peak separation) for RAS­(SD) simulations as well as from the experimental work of Vitova et al. on actinyl­(VI) systems.^52^ Data for RAS­(SD) simulations performed at experimental actinyl­(VI) bond lengths for uranyl and neptunyl taken from prior work^90^ as well as for new simulations on plutonyl, can be found in the SI and values align with trends plotted in Figure 5. The experimental 5f-σ* peak separations reported by Vitova et al. give a linear trend with a decreasing peak separation across the actinyl series. RAS­(SD) simulated spectra of uranyl­(VI) and neptunyl­(VI) follow the linear experimental peak separation trend. However, a discrepancy between simulation and experiment is found for plutonyl­(VI), with the former giving a 5f-σ* peak separation which is greater than that of neptunyl­(VI), clearly breaking the trend. The simulated actinyl­(V) peak separations follow a similar pattern to that of the actinyl­(VI) systems. The relative intensity of the 5f-σ* peaks in the simulated spectra increases across the series from U to Np in the actinyl­(VI) spectra, and from U to Pu in the actinyl­(V) spectra (Figure 2). This increasing intensity can typically be interpreted as indicating a decrease in actinide 5f-orbital mixing in the ground state 5f-σ bonding orbital. However, we note from our previous work that some caution is required when directly relating simulated peak intensity to ground state bonding character as the relationship can be complicated by orbital relaxation effects in the core-excited states and by the multiconfigurational nature of the systems.^90^ We proceed by examining the energetic relationship of the 5f-σ* peak with respect to the QTAIM data.

**Figure 5:**
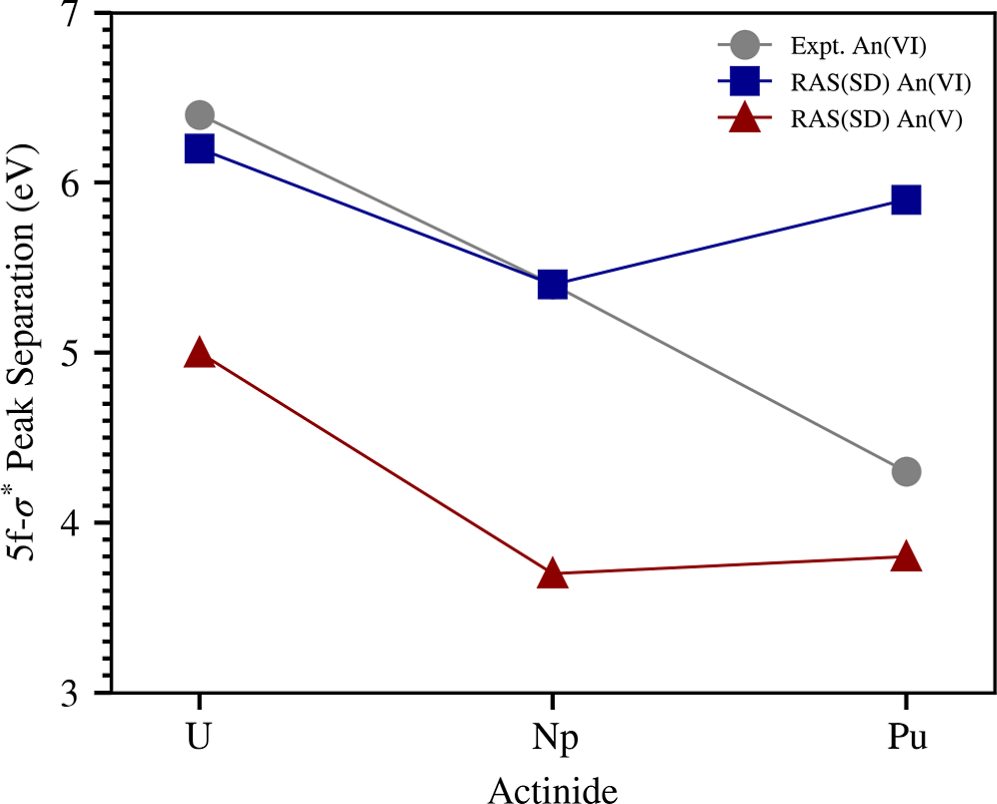
Plot of RAS­(SD) 5f-σ* peak separation values which quantify the energy difference between the position of the 5f-σ* and 5f-δ/ϕ peaks labeled in Figure 2. Energy positions used to calculate the 5f-σ* peak separation are reported in Tables S11 and S12 in the Supporting Information. Experimental (Expt.) 5f-σ* separations for actinyl­(VI) systems are taken from the work of Vitova et al.^52^

In the context of the PFB mechanism, the experimental trend for the actinyl­(VI) systems would suggest a decreasing axial An-O covalency across the series since the 5f-σ* peak separation decreases from uranyl to plutonyl. Ground-state QTAIM results reported in Table 2 counter this interpretation. The reported ρ_BCP_ values indicate that the electron density accumulation at the An–O bond critical point remains largely the same across the series. In the actinyl­(VI) systems, ρ_BCP_ values are found to increase only slightly from 0.35 to 0.38 au from uranyl to plutonyl. In actinyl­(V) systems, ρ_BCP_ values change from 0.29 to 0.31 au. The delocalization index δ­(An, O) is also reported in Table 2, and gradually increases across the series from 1.86 to 1.90 in the +6 species, and from 1.65 to 1.68 in the +5 species. Both the ρ_BCP_ and δ-values point to similar albeit weakly increasing axial covalency across the series. QTAIM analysis performed on the CESs associated with the 5f-σ* peak also finds increasing ρ_BCP_ and δ­(An, O) values across the actinyl series. These are reported in Table S13 of the SI. Based on QTAIM analysis of both the GS and relevant CESs, the trend in 5f-σ* peak separations in Figure 5 do not reflect the axial covalency across the actinyl series in the +6 or +5 oxidation state.

For a particular actinyl system, a correlation between the simulated 5f-σ* peak separation and lower covalency can be established for a change in oxidation state. Upon reduction of a given actinyl system from +6 to +5, the 5f-σ* peak separation in Figure 5 decreases substantially. For U, Np, and Pu, the 5f-σ* peak separation values reported for the An­(VI) systems drop by 1.2, 1.7, and 2.1 eV, respectively, for the An­(V) systems. This behavior has been identified in experimental studies. For instance, studies involving uranyl reduction from U­(VI) to U­(V) have shown 5f-σ* peak separations to reduce by 1.5–2.2 eV,^52,58,60^ in good agreement with the predicted value. Between Np­(VI) and Np­(V), 5f-σ* shifts have been reported to reduce by up to approximately 3.3 eV, which although greater than the predicted value of 1.7 eV in this work, represents the same qualitative behavior.^58^ The reduction in 5f-σ* peak separation from the +6 to the +5 species of a given actinyl comes with a notable decrease in both ρ_BCP_ and δ­(An, O) values by up to 0.07 au and 0.22, respectively. Therefore, the reduced An-O covalency in going from actinyl­(VI) to actinyl­(V) as indicated by the QTAIM metrics in Table 2 coincides with the reduced 5f-σ* peak separation in Figure 5. See Tables S11 and S12 of the Supporting Information for tabulated 5f-σ* shift values.

## Conclusions

Multiconfigurational simulations including spin–orbit coupling of An M_4/5_-edge XANES spectra for actinyl ions in the +6 and +5 oxidation states are presented. Simulated spectral profiles are consistent with the available experimental studies and peak assignments are in good agreement with those reported by others.^27,50,52,57,58,60^ The relative peak intensities align with the degree of 5f-character in the acceptor orbitals and the position of peaks indicate an energy ordering for the orbitals of 5f-δ/ϕ < 5f-π* < 5f-σ*. A red-shift of XANES spectra due to a reduction in actinide oxidation state has been well established in experimental studies for actinyl systems.^49,50,58,60^ In this study, we demonstrate the ability of Restricted Active Space (RAS) simulations to replicate this behavior, predicting shifts of approximately 2 eV between An­(VI) and An­(V) systems.

The shifts in An M_4/5_-edge spectra due to oxidation state arise from differences in the relative energies of the ground state (GS) and corresponding core-excited states (CESs) in the actinyl­(VI/V) ions. In all An­(VI) actinyl ions, both the GS and CESs appear at higher relative energies than in An­(V), and the resulting stabilization of the An­(V) states reflects the electron affinity of the An­(VI) species. Furthermore, the electron affinity of the An­(VI) CES is greater than that of the An­(VI) GS, resulting in an approximate 2 eV stabilization of the An­(V) CES relative to its GS. This stabilization is reflected in the lower GS→CES excitation energy in the An­(V) species, which manifests as the red-shift reproduced in the simulated XANES spectra. These relative differences in state energies are rationalized using QTAIM analysis.

By using the localization index λ_An_ from QTAIM as an indicator of variations in electron shielding between actinide centers in different states, a quantity ${\mathrm{Z̃}}_{An}$ is defined in order to track differences in the actinide effective nuclear charge. Using these metrics, the tendency of the An­(VI) species to accept an additional electron and thereby form relatively stabilized An­(V) states, arises from their greater ${\mathrm{Z̃}}_{An}$ values, which coincide with lower λ_An_ values. This indicates a greater effective nuclear charge in the An­(VI) states compared with An­(V) and accounts for their electron affinity. The differences in excitation energy for the An­(VI) and An­(V) systems can also be explained. The λ_An_ values are larger in the An­(V) GS compared to An­(VI), giving a lower effective nuclear charge in An­(V) and therefore a relatively lower 3d core–electron binding energy. This is reflected in the lower excitation energy required to access the 3d^9^ 5f^*n*+1^ CES from the 3d^10^ 5f^*n*^ GS in the An­(V) systems. Overall, the relatively high ${\mathrm{Z̃}}_{An}$ in the An­(VI) CES compared to An­(V) along with a relatively low ${\mathrm{Z̃}}_{An}$ in the An­(V) GS compared with An­(VI), gives an enhanced electron affinity in the former and reduced core–electron binding energy in the latter. The combination of these factors results in a more energetically accessible CES in the actinyl­(V) systems and thus lower excitation energy, manifesting as a red-shift in the XANES spectra. Overall, the results indicate that the electrostatic arguments commonly used to rationalize XANES shifts in terms of effective nuclear charge and core–electron binding energies are consistent with the QTAIM analysis performed on the relevant GS and CESs.

The changes in covalency for the actinyl systems was also investigated. Studies have proposed a potential link between the energy separation of the 5f-σ* and 5f-δ/ϕ peaks (5f-σ* peak separation) and actinyl axial covalency.^50,52,54,58,60,69,134,135^ Vitova et al.^52^ has reported 5f-σ* separations for the actinyl­(VI) systems which decrease linearly across the actinide series and in the context of the “pushing from below” (PFB) mechanism indicate a decreasing axial An-O covalency. The electron density at the bond critical point ρ_BCP_ and the delocalization index δ­(An, O) from QTAIM counter this interpretation and point to a weak, albeit increasing, GS covalency across the series. This is also the case for the CESs associated with the 5f-σ* peak, with increasing covalency metrics across the series. However, upon reduction of a given actinyl system from An­(VI) to An­(V), simulations predict a decrease in the 5f-σ* peak separation by 1.2, 1.7, and 2.1 eV for uranyl, neptunyl, and plutonyl, respectively. This behavior has previously been identified in experimental studies and used as an indicator for changes in axial covalency. In this study, a correlation between a decreased 5f-σ* peak separation upon actinyl reduction and lower QTAIM covalency metrics can be established, validating this feature as a marker of actinyl covalency changes due to a change in oxidation state.

Overall, results from actinyl ion simulations performed in this study demonstrate the ability of RAS calculations to correctly predict XANES shift behavior due to actinide oxidation state. The subsequent analysis of the results points to the actinide effective nuclear charge as one of the key properties for rationalizing XANES shifts. Based on the dependency of relative state energies to the effective charges, for studies aiming to predict accurate XANES shifts due to changes in actinide oxidation state, we recommend that further work consider models which include additional ligand interactions to better represent the charge distribution across entire systems. The results presented herein can therefore be considered as representative of the general behavior which can be expected when performing XANES measurements on actinyl systems.

## Supplementary Material



## Notes

Additional computational details, including active space considerations and constraints, DFT optimizations including structure coordinates, the number of states calculated in SA-RASSCF calculations and additional RAS­(SD) and RAS­(S) simulation spectra including a complete set of M_4_-and M_5_-edges not presented in the main text, assignments, and QTAIM analysis results. (PDF)

## †

Department of Chemistry, University at Buffalo State University of New York, Buffalo, New York 14260-3000, United States
