# Supplementary material for: Oxidation State Dependence of the Actinide M4/5-Edge XANES of Actinyl (An = U, Np, Pu) Systems
Source: Inorg Chem. 2026 Apr 1;65(14):7692–703. doi: 10.1021/acs.inorgchem.5c05776 (PMC13080977; doi:10.1021/acs.inorgchem.5c05776)
Supplement: Supplementary file 1 [file ic5c05776_si_001.pdf]

Supporting information for

Oxidation State Dependence of the Actinide $M_{4/5}$-edge XANES of Actinyl (An = U, Np, Pu) Systems

Kurtis Stanistreet-Welsh[a#*] and Andrew Kerridge[a*]

[a] Department of Chemistry, Lancaster University, Lancaster LA1 4YB, UK

[*] Email: kurtisst@buffalo.edu

[*] Email: a.kerridge@lancaster.ac.uk

1. Computational Details

1.1 Density Functional Theory Structure Optimisations:

Structure optimisations of $[AnO_2(H_2O)_5]^{2+/+}$ An=U, Np, Pu actinyl aquo complexes were performed using version 6.6 of TURBOMOLE.[1] The PBE0 hybrid GGA exchange-correlation functional[2,3] paired with Aldrich's def2-TZVP basis set for light elements (H,O) and def-TZVP for actinide centres (An), along with associated actinide effective core-potentials were utilised for structure optimisations.[4,5] Solvation effects outside the primary solvation sphere were modelled using an infinite dielectric continuum solvation model (COSMO) with TURBOMOLE default parameters.[6] Convergence to energetic minima structures was confirmed with vibrational frequency analysis. This choice of model chemistry has been shown previously to provide accurate actinide structure optimisations.[7-9] Based on consensus reached by previous studies,[10-14] the actinyl complexes include five water ligands to represent the primary solvation sphere and a continuum solvent model to represent the larger chemical environment. The resulting C_1 optimised structures present minimal symmetry with at least one water molecule bent out of the equatorial plane to reduce static clash between water ligands. The linearity of the An-O actinyl bond is only minimally impacted by the bounded equatorial water ligands, remaining within ~2° of a 180° O-An-O actinyl bond angle. The PBE0 actinyl penta-aquo complexes gave ground-states with spin-squared expectation values of 0.00, 0.75, and 2.02 that are consistent with a singlet $5f^0$, a doublet $5f^1$, and a triplet $5f^2$ state for uranyl, neptunyl, and plutonyl, respectively. The uranyl(V) and neptunyl(V) calculations predicted GSs with the same spin-squared expectation values as the isoelectric neptunyl(VI) and plutonyl(VI), respectively. The DFT calculations predict a plutonyl(V) open-shell $5f^3$ quartet GS with a spin-expectation value of 3.79 compared to the formal value of 3.75 for a pure quartet spin-state, indicating only marginal spin-contamination. These DFT predicted GS multiplicities are consistent with the more extensive actinyl GS study of Hay et al. and therefore considered appropriate to inform the bond lengths used in restricted active space calculations.[10]

1.2 RASSCF and SS-RASPT2 Details:

Scalar relativistic Restricted Active Space Self-Consistent Field (RASSCF) calculations were performed on bare actinyl complexes $[AnO_2]^{2+/+}$ An=U, Np, Pu using version 21.02 of Openmolcas.[15-18] Actinyl models were constrained to D_{2h} symmetry with bond lengths set to those obtained from the PBE0 optimised actinyl aquo complexes detailed in section 1.1. A separate $[PuO_2]^{2+}$ calculation was performed with a experimentally informed Pu-O bond length (1.74Å) to enable a comparison with previously reported results for uranyl and neptunyl.[19,20] All-electron relativistic ANO-RCC TZVP basis sets of Roos et al.[21;22] were employed for An (basis functions: 9s8p6d4f2g) and O (basis functions: 4s3p2s1f) centres with higher angular momentum h-functions removed from actinides to enable compatibility with analysis software. Scalar relativistic effects were modelled using the second order Douglas-Kroll-Hess Hamiltonian and Cholseky decomposition was utilised throughout to speed-up integral calculations.[23-26]

State-average (SA) RASSCF calculations were performed to obtain the necessary number of ground- and core-excited states to simulate the XANES spectra, with details of both the active space and state-average calculations outlined in the proceeding sections (1.3 and 1.4). State-specific 2nd order RAS perturbation theory[27;28] with a default IPEA shift[29] of 0.25 a.u. and imaginary shift[30] of 0.5 a.u. were performed on RASSCF states to recover dynamical correlation and gain quantitative state energies. The chosen imaginary shift value offered a reasonable balance between converging intruder free solutions without introducing a significant bias to the resulting RASPT2 energies, and are comparable to values used in related studies.[31-36]

1.3 Actinyl M-edge XANES Active Space Details:

The active space used to calculate the ground state (GS) and core-excited states (CESs) needed for actinyl XANES simulations is presented in figure 1 of the main text. The active space includes: the g-parity 3d core-orbitals ($5 \times 3d$) spanning RAS1, the u-parity bonding orbitals ($2 \times \pi_u$, $1 \times \sigma_u$) spanning RAS2, and the u-parity anti-bonding orbitals ($2 \times \pi_u^*$, $1 \times \sigma_u^*$) spanning RAS3. The non-bonding 5f orbitals are also included within RAS3 ($2 \times 5f_\delta$, $2 \times 5f_\phi$) as a means by which to manage calculation cost. Built-in supersymmetry designations of Openmolcas were utilised to restrict the rotation of core-orbitals out of the active space during the SCF procedure.

The RASSCF calculations are split into two levels of quality, denoted RAS(S) and RAS(SD), consistent with our previous work.[19;20] The results from RAS(SD) calculations, which form the basis of the main text, are presented first. The RASSCF active-spaces and constraints are written in terms of Sauri notation[37] as RAS(a,*b*,*c*;*i*,*j*,*k*), where i, j, and k denote the active orbitals in RAS1, RAS2 and RAS3, respectively. The indices *a*, *b*, and *c* represent the total number of active electrons, the maximum number of holes allowed in RAS1, and the maximum number of electrons allowed in RAS3, respectively.

For RAS(SD) simulations performed in this study, i=5, j=3, and k=7, corresponding to the five 3d core orbitals placed in RAS1, the three bonding orbitals in RAS2, and the seven total non-bonding and anti-bonding orbitals in RAS3. This active space is shown in figure 1 of the main text. For all actinyl simulations, the total number of active electrons is a minimum of a=16, accounting for the 10 electrons in the core 3d orbitals (RAS1) and 6 electrons in the bonding orbitals (RAS2).

In their ground states, actinyl ions typically adopt configurations in which *n* unpaired electrons occupy the non-bonding 5f orbitals, giving $5f^n$ states. Uranyl(VI), neptunyl(VI) and plutonyl(VI) correspond to $5f^0$, $5f^1$ and $5f^2$ states, respectively, whereas uranyl(V), neptunyl(V) and plutonyl(V) form $5f^1$, $5f^2$ and $5f^3$ states. Therefore, for any actinyl system, the total number of active electrons is given by a=16+n. Specifically, a=16 for U(VI), 17 for Np(VI) and U(V), 18 for Pu(VI) and Np(V), and 19 for Pu(V).

The non-bonding 5f orbitals are placed in RAS3, and the lower bound on RAS3 occupation is fixed at *n*, ensuring that the calculation can represent the correct $5f^n$ configurations which arise in the GS. At the RAS(SD) level, RAS3 is allowed to contain up to two additional electrons relative to the n electrons already allowed for $5f^n$ configurations, giving an upper bound of c=n+2 in RAS3. This gives c=2 for U(VI), 3 for Np(VI) and U(V), 4 for Pu(VI) and Np(V), and 5 for Pu(V).

Overall, the ground-state RAS(SD) calculations can be expressed as RAS(16+n,0,n+2;5,3,7), and the corresponding core excited state calculations as RAS(16+n,1,n+2;5,3,7), where one hole is enforced in RAS1 (b=1).

For RAS(S) level calculations, a modestly expanded active space is employed in which the g-parity valence bonding orbitals ($2 \times \pi_g$, $1 \times \sigma_g$) are included in RAS2. These calculations and their results are presented in the Supporting Information for interested readers and serve primarily as a guide for XANES peak assignment. However, it should be noted that these simulations give a good qualitative representation of the spectra and the overall results are consistent with the more computationally costly RAS(SD). At the RAS(S) level, only one additional electron beyond the $5f^n$ occupation is permitted to enter RAS3. The corresponding active-space definitions for the ground state and core-excited states are therefore RAS(16+n,0,n+1;5,6,7) and RAS(16+n,1,n+1;5,6,7), respectively.

Further details on the state-average calculations used to generate the required number of ground- and core-excited states for XANES simulations are provided in section 1.4.

1.4 State-Average and RASSI calculation Details:

For the purposes of this study, RASSCF states corresponding to different distributions of electrons among the non-bonding 5f orbitals are taken to represent the possible ground-state (GS) configurations prior to inclusion of spin–orbit coupling. The RASSCF states with core-holes are classified as core-excited states (CES). The appropriate number of GS and CESs for each system obtained via state-average (SA)-RASSCF calculations are reported in table S1. The number of these states which are further spin-orbit coupled *post hoc via* state-interaction with a mean-field spin-orbit operator in the Restricted Active Space State Interaction (RASSI) formalism is also reported.[38;39] The requested number of GSs and CESs from SA-RASSCF calculations is conveniently broken down into irrep and spin-multiplicity. For RAS(S) calculations, the number of RASSCF states calculated by performing a state-average correspond to the total number of possible ground- and core-excited states of each spin-multiplicity and irrep and were all supplied to RASSI calculations.

Table S1. Number and type of RAS(S) state-averaged calculations performed to generate the ground- and core-excited states supplied to RASSI for XANES simulations of the actinyl systems.

Simulation	Set of States: $^{Spin\text{-}Mult}$(Irreps)	SA-RASSCF	Included in RASSI
U(VI) M_4	GS: $^1(A_g)$	5	1
	CES: $^1(A_u,B_{1u},B_{2u},B_{3u})$	8,9,9,9	8,9,9,9
	CES: $^3(A_u,B_{1u},B_{2u},B_{3u})$	8,9,9,9	8,9,9,9
Np(VI) M_5	GS: $^2(A_u,B_{1u},B_{2u},B_{3u})$	5,5,5,5	1,1,1,1
	CES: $^2(A_g,B_{1g},B_{2g},B_{3g})$	62, 61, 61, 61	62, 61, 61, 61
	CES: $^4(A_g,B_{1g},B_{2g},B_{3g})$	24, 27, 27, 27	24, 27, 27, 27
Pu(VI) M_5	GS: $^1(A_g,B_{1g},B_{2g},B_{3g})$	5,5,5,5	1,1,1,1
	GS: $^3(A_g,B_{1g},B_{2g},B_{3g})$	5,5,5,5	1,1,1,1
	CES: $^1(A_u,B_{1u},B_{2u},B_{3u})$	140,140,140,140	140,140,140,140
	CES: $^3(A_u,B_{1u},B_{2u},B_{3u})$	186,183,183,183	186,183,183,183
	CES: $^5(A_u,B_{1u},B_{2u},B_{3u})$	46,43,43,43	46,43,43,43
U(V) M_4	GS: $^2(A_u,B_{1u},B_{2u},B_{3u})$	5,5,5,5	1,1,1,1
	CES: $^2(A_g,B_{1g},B_{2g},B_{3g})$	62, 61, 61, 61	62, 61, 61, 61
	CES: $^4(A_g,B_{1g},B_{2g},B_{3g})$	24, 27, 27, 27	24, 27, 27, 27
Np(V) M_5	GS: $^1(A_g,B_{1g},B_{2g},B_{3g})$	5,5,5,5	1,1,1,1
	GS: $^3(A_g,B_{1g},B_{2g},B_{3g})$	5,5,5,5	1,1,1,1
	CES: $^1(A_u,B_{1u},B_{2u},B_{3u})$	140,140,140,140	140,140,140,140
	CES: $^3(A_u,B_{1u},B_{2u},B_{3u})$	186,183,183,183	186,183,183,183
	CES: $^5(A_u,B_{1u},B_{2u},B_{3u})$	46,43,43,43	46,43,43,43
Pu(V) M_5	GS: $^2(A_u,B_{1u},B_{2u},B_{3u})$	5,5,5,5	1,1,1,1
	GS: $^4(A_u,B_{1u},B_{2u},B_{3u})$	5,5,5,5	1,1,1,1
	CES: $^2(A_g,B_{1g},B_{2g},B_{3g})$	512,506,506,506	512,506,506,506
	CES: $^4(A_g,B_{1g},B_{2g},B_{3g})$	304,307,307,307	304,307,307,307
	CES: $^6(A_g,B_{1g},B_{2g},B_{3g})$	46,43,43,43	46,43,43,43

For RAS(SD) simulations, state-averaged calculations were carried out with the Opnemolcas default 600 state maximum per spin multiplicity and irrep cap in-mind, both to aid ease of reproducibility and simply to limit computational cost of the subsequent RASSI step. This level of state averaging was found to be sufficient for capturing states within the spectral range of interest for generating a XANES spectrum. RAS(S) simulations which include all the possible core-excited states which can be generated in the RASSI step (within the Laporte selection rule) were performed prior to RAS(SD) to give an indication of the energy range in which the main spectral features fall and used to inform the state calculations at the RAS(SD) level. The results of RAS(S) simulations can be viewed in figures S21 and S22. The RASSI calculations constitute the primary bottleneck for XANES simulations and therefore particular care was taken to reduce the number of states passed to the RASSI procedure in order to keep the RAS(SD) level simulations tractable. To restrict the number of states supplied to RASSI, the following criteria were applied for state selection:

1. The Laporte selection rule was applied at the SA-RASSCF level to restricted the number of ground- and core-excited states that need to be computed. For example, the uranyl GS belongs to a g-parity irrep, so only u-parity CESs were required. This substantially reduces the number of states entering the RASSI step.
2. From the set of scalar-relativistic (spin-free) RASSCF states obtained from state-average calculations, only those below a chosen energy cutoff were supplied to RASSI. These cutoffs were determined by inspecting spin-free RAS(S) XANES spectra (computed prior to RAS(SD) simulations) and identifying the energy at which all spectral features of interest terminate. This procedure effectively reduces the number of states entering the spin-orbit coupled RASSI calculations while preserving all states relevant to experimental spectral features. The cutoffs employed for RAS(SD) simulations were 3655, 3771 and 3886 eV for uranyl(VI), neptunyl(VI) and plutonyl(VI), respectively, and 3651, 3767, and 3884 eV for uranyl(V), neptunyl(V) and plutonyl(V).
3. To further reduce RASSI costs, the spin-orbit ground states were pre-optimised such that only the dominant contributing spin-free RASSCF states were retained. This avoids unnecessary state interactions and ensures that only those states essential for an accurate description of the spin-orbit ground state are included in the RASSI procedure.

Note that even with these criteria, the RAS(SD) calculation for $[Pu(V)O_2]^+$ required state interaction over 6240 spin-free states, yielding 23044 spin–orbit states in the subsequent RASSI step. These calculations demanded substantial computational resources, with wall times on the order of several weeks, and would be impracticable at present without the state-pruning strategy employed here.

Table S2. Number and type of RAS(SD) state-averaged calculations performed to generate the ground- and core-excited states supplied to RASSI for XANES simulations of the actinyl systems.

Simulation	Set of States: $^{Spin\text{-}Mult}$(Irrep)	SA-RASSCF	Included in RASSI
U(VI) M_4	GS: $^1(A_g)$	5	1
	CES: $^1(A_u,B_{1u},B_{2u},B_{3u})$	191,193,193,193	147,147,147,147
	CES: $^3(A_u,B_{1u},B_{2u},B_{3u})$	272,271,271,271	225,218,211,211
Np(VI) M_5	GS: $^2(A_u,B_{1u},B_{2u},B_{3u})$	5,5,5,5	1,1,1,1
	CES: $^2(A_g,B_{1g},B_{2g},B_{3g})$	597,598,599,599	597,598,599,599
	CES: $^4(A_g,B_{1g},B_{2g},B_{3g})$	599,597,596,596	490,493,476,476
Pu(VI) M_5	GS: $^1(A_g,B_{1g},B_{2g},B_{3g})$	5,5,5,5	0,1,0,0
	GS: $^3(A_g,B_{1g},B_{2g},B_{3g})$	5,5,5,5	1,0,1,1
	CES: $^1(A_u,B_{1u},B_{2u},B_{3u})$	600,600,600,600	465,455,462,462
	CES: $^3(A_u,B_{1u},B_{2u},B_{3u})$	594,598,600,600	594,598,600,600
	CES: $^5(A_u,B_{1u},B_{2u},B_{3u})$	597,600,596,596	449,444,462,462
U(V) M_4	GS: $^2(A_u,B_{1u},B_{2u},B_{3u})$	5,5,5,5	1,1,1,1
	CES: $^2(A_g,B_{1g},B_{2g},B_{3g})$	598,597,597,600	463,463,473,473
	CES: $^4(A_g,B_{1g},B_{2g},B_{3g})$	600,600,599,600	365,367,359,365
Np(V) M_5	GS: $^1(A_g,B_{1g},B_{2g},B_{3g})$	5,5,5,5	0,1,0,0
	GS: $^3(A_g,B_{1g},B_{2g},B_{3g})$	5,5,5,5	1,0,1,1
	CES: $^1(A_u,B_{1u},B_{2u},B_{3u})$	600,600,597,597	407,390,404,404
	CES: $^3(A_u,B_{1u},B_{2u},B_{3u})$	600,599,599,593	600,599,599,593
	CES: $^5(A_u,B_{1u},B_{2u},B_{3u})$	600,600,600,600	425,427,424,424
Pu(V) M_5	GS: $^2(A_u,B_{1u},B_{2u},B_{3u})$	5,5,5,5	0,0,0,0
	GS: $^4(A_u,B_{1u},B_{2u},B_{3u})$	5,5,5,5	1,1,1,1
	CES: $^2(A_g,B_{1g},B_{2g},B_{3g})$	599,600,599,599	599,600,599,599
	CES: $^4(A_g,B_{1g},B_{2g},B_{3g})$	600,600,600,600	600,600,600,600
	CES: $^6(A_g,B_{1g},B_{2g},B_{3g})$	600,596,598,598	397,352,345,345

Table S3. Number and type of RAS(SD) state-averaged calculations performed to obtain the states required for Pu M_5-edge simulations of plutonyl set to the experimental bond length of 1.74Å.[10;40] An energy cutoff of 3885 eV was used to determine which states were included in the RASSI step.

Simulation	Set of States: $^{Spin\text{-}Mult}$(Irrep)	SA-RASSCF	Included in RASSI
Pu(VI) M_5 Expt. Bond Length	GS: $^1(A_g,B_{1g},B_{2g},B_{3g})$	5,5,5,5	1,1,1,1
	GS: $^3(A_g,B_{1g},B_{2g},B_{3g})$	5,5,5,5	1,1,1,1
	CES: $^1(A_u,B_{1u},B_{2u},B_{3u})$	600,600,600,600	405,411,384,384
	CES: $^3(A_u,B_{1u},B_{2u},B_{3u})$	595,598,600,600	595,598,600,600
	CES: $^5(A_u,B_{1u},B_{2u},B_{3u})$	599,600,599,599	408,411,411,411

2. Peak Broadenings:

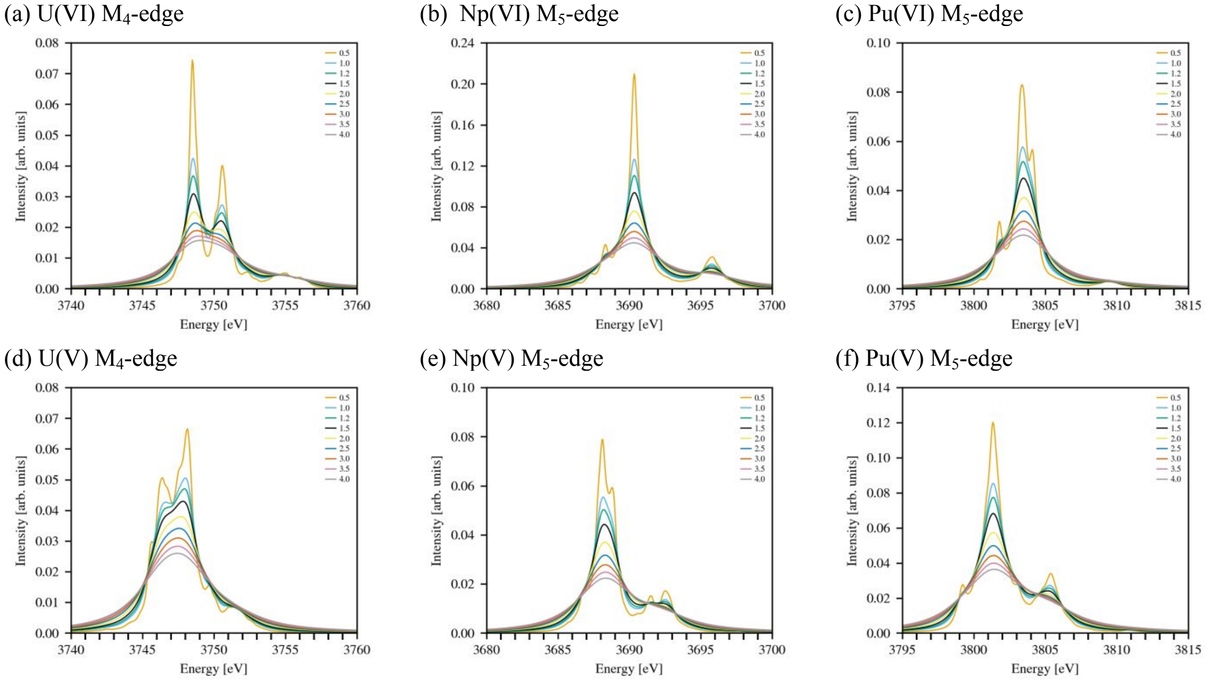

Figure S1. (a) U(VI) M_4, (b) Np(VI) M_5, (c) Pu(VI) M_5, (d) U(V) M_4, (e) Np(V) M_5, and (f) Pu(V) M_5-edge XANES RAS(SD) spectra. Plots show various FWHM values utilized in the Lorentzian broadening of RAS(SD) transitions.

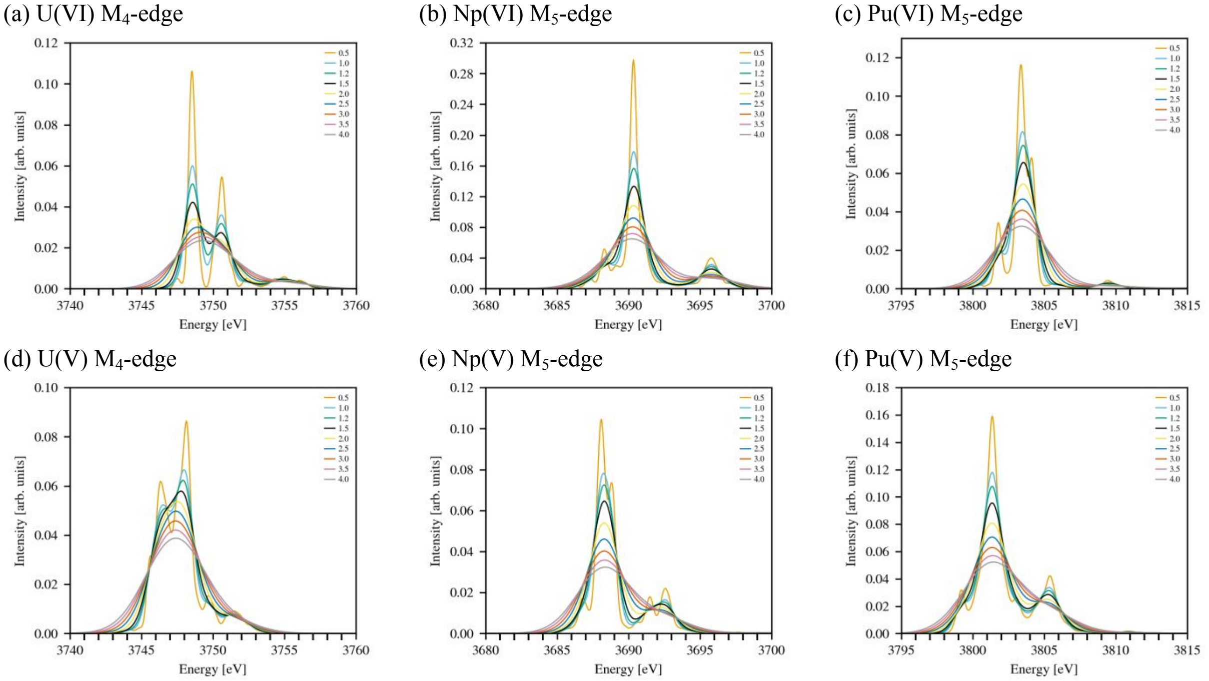

Figure S2. (a) U(VI) M_4, (b) Np(VI) M_5, (c) Pu(VI) M_5, (d) U(V) M_4, (e) Np(V) M_5, and (f) Pu(V) M_5-edge XANES RAS(SD) spectra. Plots show various FWHM values utilized in the Gaussian broadening of RAS(SD) transitions

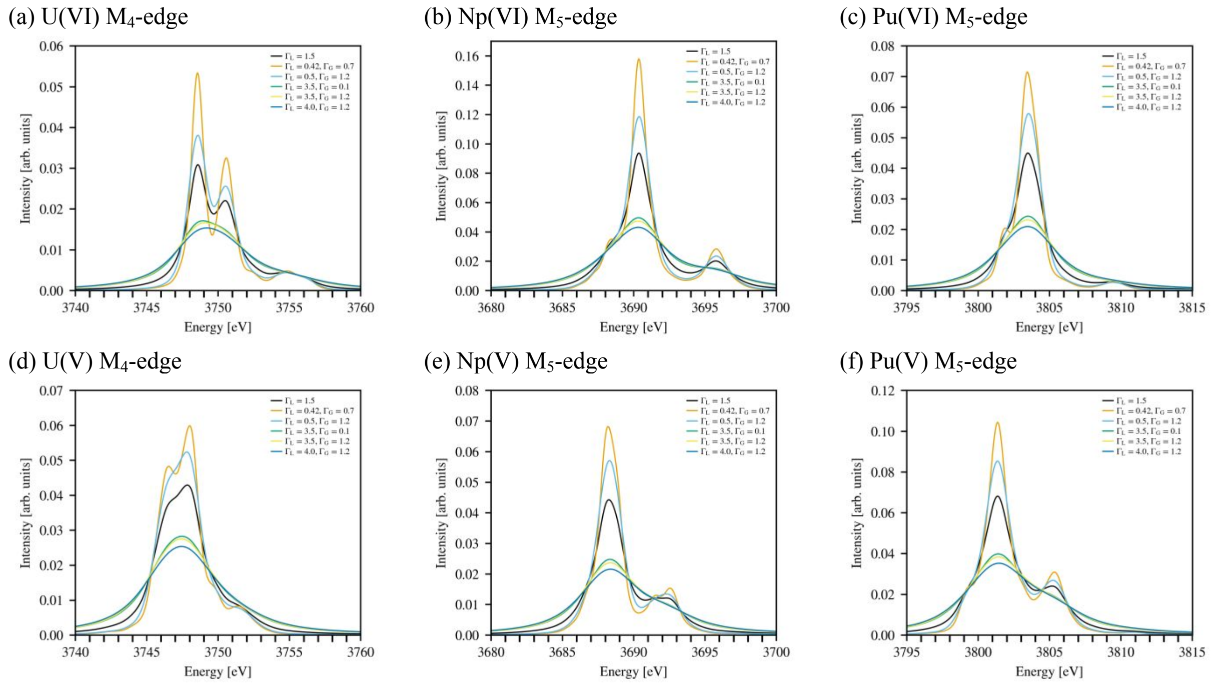

Figure S3. (a) U(VI) M_4, (b) Np(VI) M_5, (c) Pu(VI) M_5, (d) U(V) M_4, (e) Np(V) M_5, and (f) Pu(V) M_5-edge XANES RAS(SD) spectra. Spectra are plotted using various FWHM values for the Lorentzian (Γ_L) and Gaussian (Γ_L) contributions in the Voigt function. A plot obtained using Lorentzian broadening with a FWHM of 1.5 (Γ_L=1.5) is included and corresponds the broadening utilized in the main manuscript.

Figures S1-S3 present the application of three broadening schemes, Lorentzian, Gaussian and Voigt, used to broaden simulated transitions into XANES spectra. For each scheme, a range of full-width at half maximum (FWHM) values is explored and the resulting spectra are presented. These broadening approaches have been previously employed in $M_{4/5}$-edge XANES simulations of actinyl(VI) systems.[19;20;31;36;40-42] Regardless of the broadening scheme employed, the energy positions of the main spectral features remain unchanged, and overall spectral profiles are qualitatively similar.

In figure S3, spectra obtained using Voigt broadening are presented alongside spectra obtained using Lorentzian broadening with a FWHM of 1.5 eV, which is the value employed for the main manuscript figures and the remainder of this Supporting Information. The Voigt broadening scheme provides a convenient framework for separating contributions associated with the intrinsic core-hole lifetime, represented by the Lorentzian component, from those arising from experimental resolution effects, represented by the Gaussian component.

Figure S1 demonstrates that employing Lorentzian FWHM values in the range of 3-4 eV, consistent with reported $M_{4/5}$-edge core-hole lifetimes for actinides,[43-45] results in very broad XANES spectra with limited peak structure. When these Lorentzian FWHM values are used within a Voigt scheme, the resulting spectra remain broad irrespective of the Gaussian contribution. In contrast, Lorentzian FWHM values below ~2 eV are required to reproduce the spectral resolutions observed in high-resolution XANES measurements. For example, Vitova et al.[40] obtained XANES spectra from slices of resonant inelastic X-ray scattering (RIXS) maps, where the effective spectral resolution is no longer strictly limited by the core-hole lifetime. Polly et al.[36] modelled both RIXS maps, incorporating ground, intermediate, and final states, as well as high-resolution (HR) XANES spectra that include only the ground and core-excited states, consistent with the approach adopted in the present study. In that work, the RIXS-derived XANES slices are well reproduced by the HR-XANES simulations using a Lorentzian broadening of 1.5 eV, which also compared well with the XANES spectrum reported by Vitova et al.

We further examine Voigt broadenings using FWHM values reported in studies that aim to directly reproduce specific experimental XANES spectra. Here, Lorentzian FWHM values of 0.5 or 0.42 eV in combination with Gaussian FWHM values of 1.2 or 0.7 eV, respectively, are employed.[40;42] Testing these parameters yields spectra that are qualitatively similar to those obtained using Lorentzian broadening alone.

To maintain consistency with prior RAS-based XANES simulations of actinyl systems[19;20;36;41] and because the present work does not seek to reproduce the profiles of a specific experimental spectrum, Lorentzian broadening is employed to generate representative XANES profiles characteristic of high-resolution measurements. A Lorentzian FWHM of 1.5 eV, as reported by Polly et al.,[36] is therefore used throughout this study. For future work aimed at quantitative comparison with specific experimental spectra, Voigt broadening offers additional flexibility for independently accounting for intrinsic lifetime effects and experimental resolution.

3. DFT Optimised Structures:

PBE0 optimised structures (C_1).

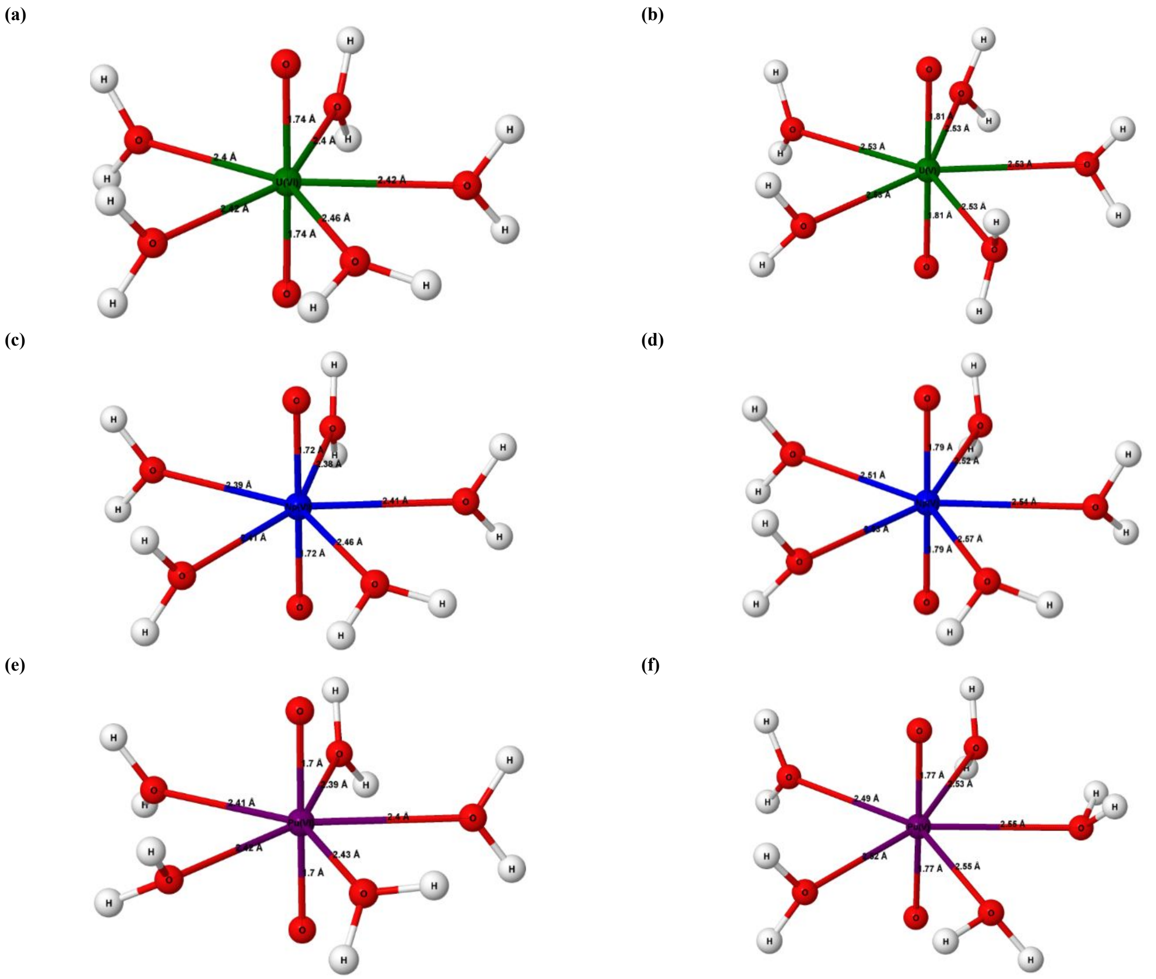

Figure S4. C_1 PBE0 $[AnO_2(H_2O)_5]^{2+/+}$ optimised structures. Actinyl An-O bond lengths were utilized for D_2h $[AnO_2]^{2+/+}$ RASSCF simulations. The structures of (a) U(VI), (b) U(V), (c) Np(VI), (d) Np(V), (e) Pu(VI), and (f) Pu(V) are presented.

Table S4. PBE0 $[AnO_2(H_2O)_5]^{2+/+}$ axial (ax.) An-O bond lengths (Å) for the actinyl unit and equatorial (eq.) An-OH_2 bond lengths (Å) for coordinating water ligands.

System	An-O (ax.)	Average An-OH_2 (eq.)
$[UO_2(H_2O)_5]^{2+}$	1.74	2.42
$[NpO_2(H_2O)_5]^{2+}$	1.72	2.41
$[PuO_2(H_2O)_5]^{2+}$	1.70	2.41
$[UO_2(H_2O)_5]^{+}$	1.81	2.53
$[NpO_2(H_2O)_5]^{+}$	1.79	2.53
$[PuO_2(H_2O)_5]^{+}$	1.77	2.53

Table S5. PBE0 $[AnO_2(H_2O)_5]^{2+/+}$ axial (ax.) An-O bond angles (degrees) for the actinyl unit.

System	∡(O-An-O)
$[UO_2(H_2O)_5]^{2+}$	177.9
$[NpO_2(H_2O)_5]^{2+}$	178.7
$[PuO_2(H_2O)_5]^{2+}$	179.1
$[UO_2(H_2O)_5]^{+}$	179.9
$[NpO_2(H_2O)_5]^{+}$	178.8
$[PuO_2(H_2O)_5]^{+}$	178.9

Table S6. Experimental[10;46] and PBE0 $[AnO_2(H_2O)_5]^{2+/+}$ axial (ax.) An-O bond lengths (Å) for the actinyl unit as well as Shannon ionic radii for the actinide-ions in the +6 and +5 states.[41] All values reported in angstroms (Å).

System	Experimental An-O (ax.)	An-O (ax.)	Ionic Radius
$[UO_2(H_2O)_5]^{2+}$	1.76	1.74	0.73
$[NpO_2(H_2O)_5]^{2+}$	1.75	1.72	0.72
$[PuO_2(H_2O)_5]^{2+}$	1.74	1.70	0.71
$[UO_2(H_2O)_5]^{+}$		1.81	0.76
$[NpO_2(H_2O)_5]^{+}$		1.79	0.75
$[PuO_2(H_2O)_5]^{+}$		1.77	0.74

Comment on relationship between ionic radius and bond lengths: Table S6 reports the experimental and PBE0 derived actinyl bond lengths along with Shannon ionic radii.[41] The change in experimental bond lengths (0.01Å) across the series can be explained by a decrease actinide ionic radius which decrease by 0.01Å across the actinyl series. Since the theoretical bond lengths of the An(V) actinyls change by the same trend as the An(VI) actinyls, these too are suspected to be explained by changes in ionic radii.

Table S7. PBE0 $[An^{VI}O_2(H_2O)_5]^{2+}$ optimised structure XYZ coordinates

$[UO_2(H_2O)_5]^{2+}$				$[NpO_2(H_2O)_5]^{2+}$				$[PuO_2(H_2O)_5]^{2+}$			
Atom	x	Y	z	Atom	x	y	z	Atom	x	y	z
U	-0.0468	-0.0146	-0.03158	Np	0.039258	0.03414	0.032886	Pu	-0.00566	-0.0011	-0.00258
O	-0.22252	-0.06699	1.699494	O	-0.15261	0.097883	1.738	O	-0.1577	-0.04192	1.69154
O	0.068915	0.014382	-1.7684	O	0.261376	-0.00337	-1.66932	O	0.133139	0.017321	-1.69804
O	-2.3309	0.698584	-0.21679	O	-2.30004	0.502463	-0.3048	O	-2.28565	0.758713	-0.15346
H	-2.80972	0.863951	-1.03718	H	-2.6712	0.819606	-1.13662	H	-2.68203	1.10053	-0.96339
O	-1.29257	-2.06076	-0.17937	O	-1.34633	-2.00095	-0.01425	O	-1.32615	-1.98513	-0.13191
H	-1.6548	-2.56366	0.559181	H	-0.93061	-2.84892	-0.20889	H	-1.47636	-2.61576	0.581456
O	1.613461	-1.77474	0.085992	O	1.345733	-1.97791	0.236855	O	1.603418	-1.78495	0.06634
H	1.764186	-2.3267	0.862879	H	1.685415	-2.31026	1.076548	H	1.94013	-2.18464	0.87681
O	2.250259	0.855013	0.138853	O	2.290103	0.779102	0.277747	O	2.23597	0.927739	0.146401
H	2.983712	0.29997	0.427211	H	2.8854	1.019088	-0.44173	H	3.001357	0.357307	0.008769
O	0.034014	2.40472	0.060808	O	-0.1209	2.41299	-0.03858	O	-0.02352	2.413365	0.083192
H	-0.228	2.923248	0.831113	H	-0.37155	2.978041	0.701435	H	-0.76245	2.898383	0.469106
H	-2.95737	0.816571	0.506593	H	-2.91254	0.766988	0.391829	H	-3.00352	0.378783	0.366538
H	-1.50782	-2.54395	-0.98555	H	-2.20259	-1.99257	-0.45716	H	-1.74214	-2.3471	-0.92252
H	1.945538	-2.26824	-0.67361	H	1.942689	-2.29853	-0.45014	H	1.7192	-2.43773	-0.63398
H	2.43451	1.75681	0.425292	H	2.706866	1.06369	1.099367	H	2.483373	1.57007	0.821709
H	-0.04409	2.986396	-0.70494	H	-0.14846	2.958527	-0.83316	H	0.348584	2.976113	-0.60598

Table S8. PBE0 $[An^{V}O_2(H_2O)_5]^+$ optimised structure XYZ coordinates

$[UO_2(H_2O)_5]^+$				$[NpO_2(H_2O)_5]^+$				$[PuO_2(H_2O)_5]^+$			
Atom	x	y	z	Atom	x	y	z	Atom	x	y	Z
U	0.001488	-0.01004	0.000905	Np	0.040968	0.006811	-0.0039	Pu	-0.05328	-0.00325	0.005364
O	0.011472	1.798901	0.006175	O	-0.14809	0.08353	1.769771	O	-0.10512	1.760693	0.041296
O	-0.01024	-1.81895	-0.00298	O	0.251836	-0.03948	-1.77857	O	-0.02304	-1.76797	-0.00612
O	-2.25824	-0.01594	-1.12895	O	-2.41441	0.522853	-0.3083	O	-2.19721	-0.06406	-1.31826
H	-2.52992	-0.77736	-1.65275	H	-2.7404	1.005214	-1.07535	H	-2.76313	-0.84335	-1.32487
O	-1.773	-0.01982	1.802967	O	-1.42042	-2.10352	0.011984	O	-1.69308	-0.06253	1.874509
H	-2.26783	0.786192	1.986347	H	-0.95623	-2.91079	-0.23597	H	-1.95266	0.699339	2.402405
O	1.159888	-0.02152	2.249815	O	1.370147	-2.14243	0.250427	O	1.477498	0.03736	2.023991
H	1.197971	0.787254	2.771698	H	1.695856	-2.39667	1.12064	H	1.484428	0.796058	2.617297
O	2.495552	-0.03005	-0.4079	O	2.437351	0.718676	0.27798	O	2.421313	0.091774	-0.61025
H	3.023996	-0.79009	-0.14198	H	2.91683	1.146452	-0.43934	H	2.937699	0.047102	0.203667
O	0.3819	0.005264	-2.49985	O	-0.05276	2.512797	-0.04437	O	0.381922	0.014379	-2.50851
H	0.719421	0.798601	-2.92932	H	-0.43376	3.031451	0.671606	H	0.902451	0.741516	-2.86673
H	-2.56722	0.758065	-1.61204	H	-2.98003	0.782586	0.426574	H	-2.78992	0.694082	-1.35895
H	-2.41951	-0.73373	1.828861	H	-2.22036	-2.07436	-0.52395	H	-1.91227	-0.84068	2.397001
H	0.972023	-0.72822	2.877107	H	2.090327	-2.33073	-0.36115	H	1.57188	-0.73777	2.588032
H	3.035285	0.745174	-0.21975	H	2.730725	1.151982	1.086439	H	2.771177	-0.60298	-1.17858
H	0.826957	-0.73373	-2.92835	H	-0.16757	3.035625	-0.84453	H	-0.45866	0.040291	-2.98128

4. RAS(SD) Additional Results

Additional XANES spectra:

(a) (b)

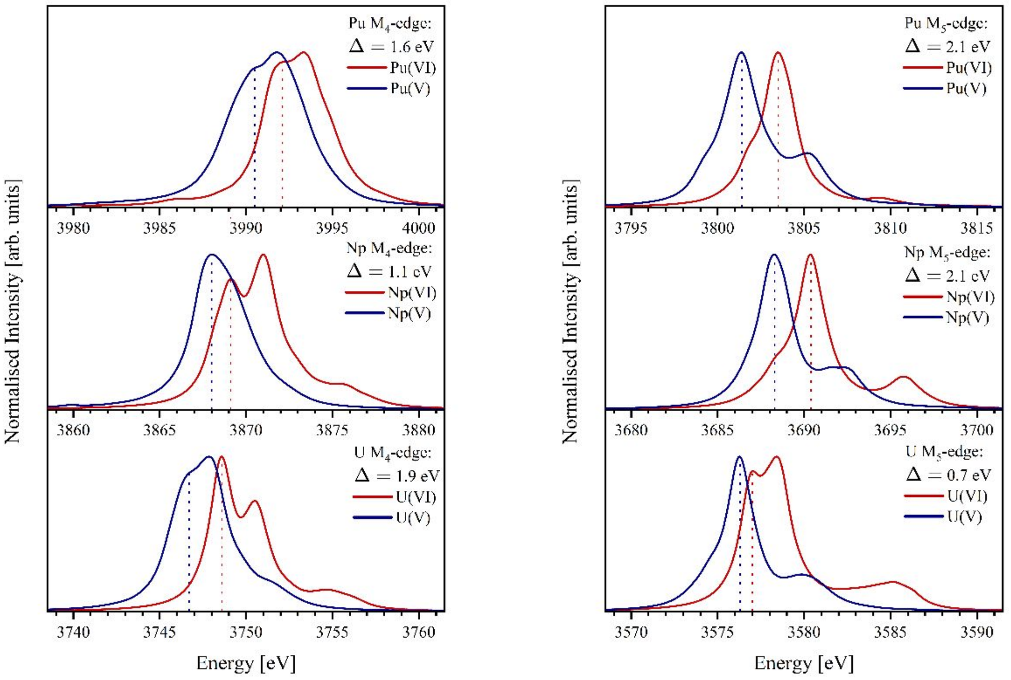

Figure S5. Additional RAS(SD) (a) M_4-edge and (b) M_5-edge XANES spectra for actinyls(VI/V).

Overall Assignments:

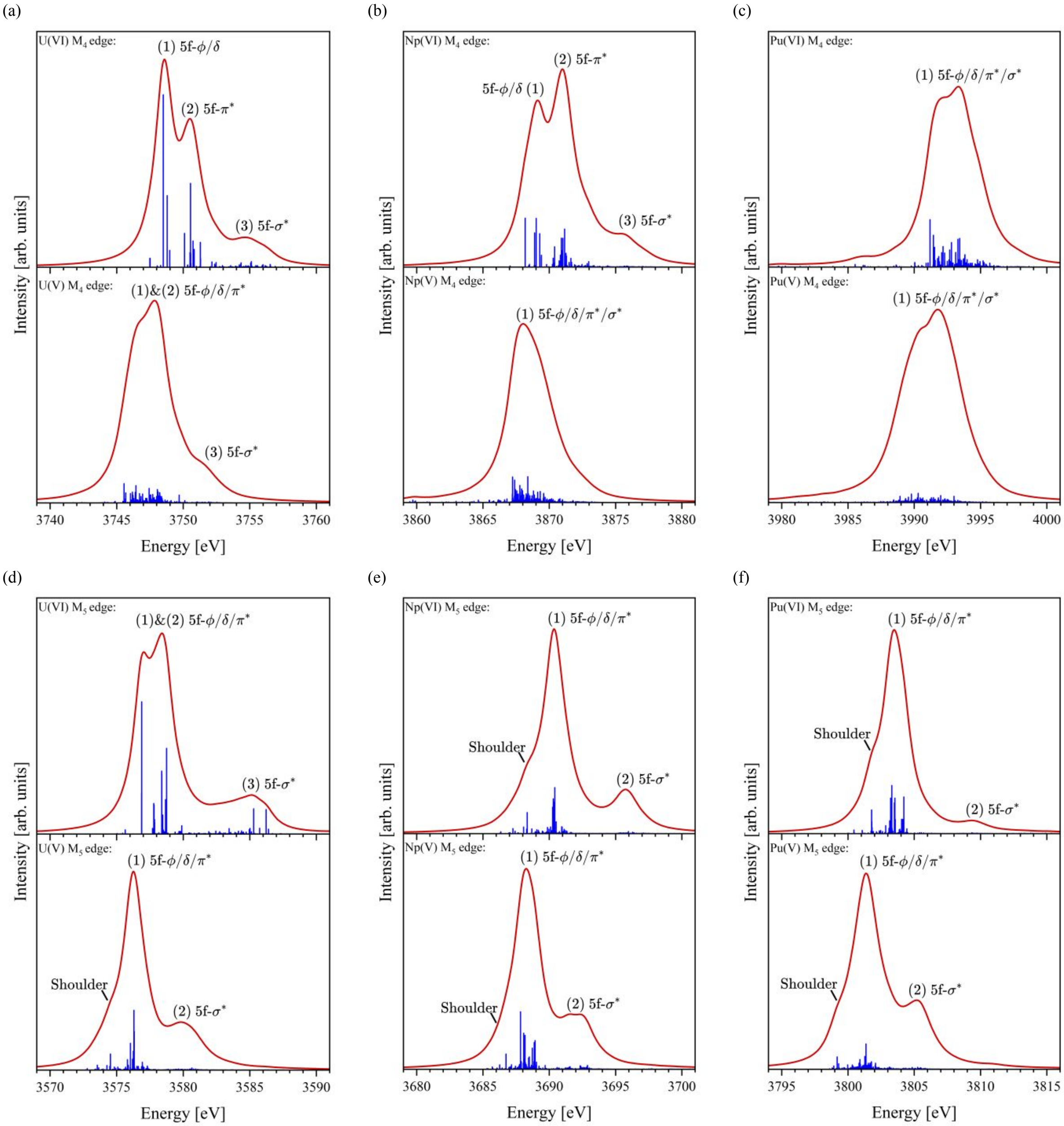

Figure S6. Separate RAS(SD) (a) U M_4-, (b) Np M_4-, (c) Pu M_4-, (d) U M_5-, (e) Np M_5-, and (f) Pu M_5-edge XANES spectra. Each plot presents overall peak assignments for actinyl(VI) and actinyl(V) systems.

State-Energy Diagrams

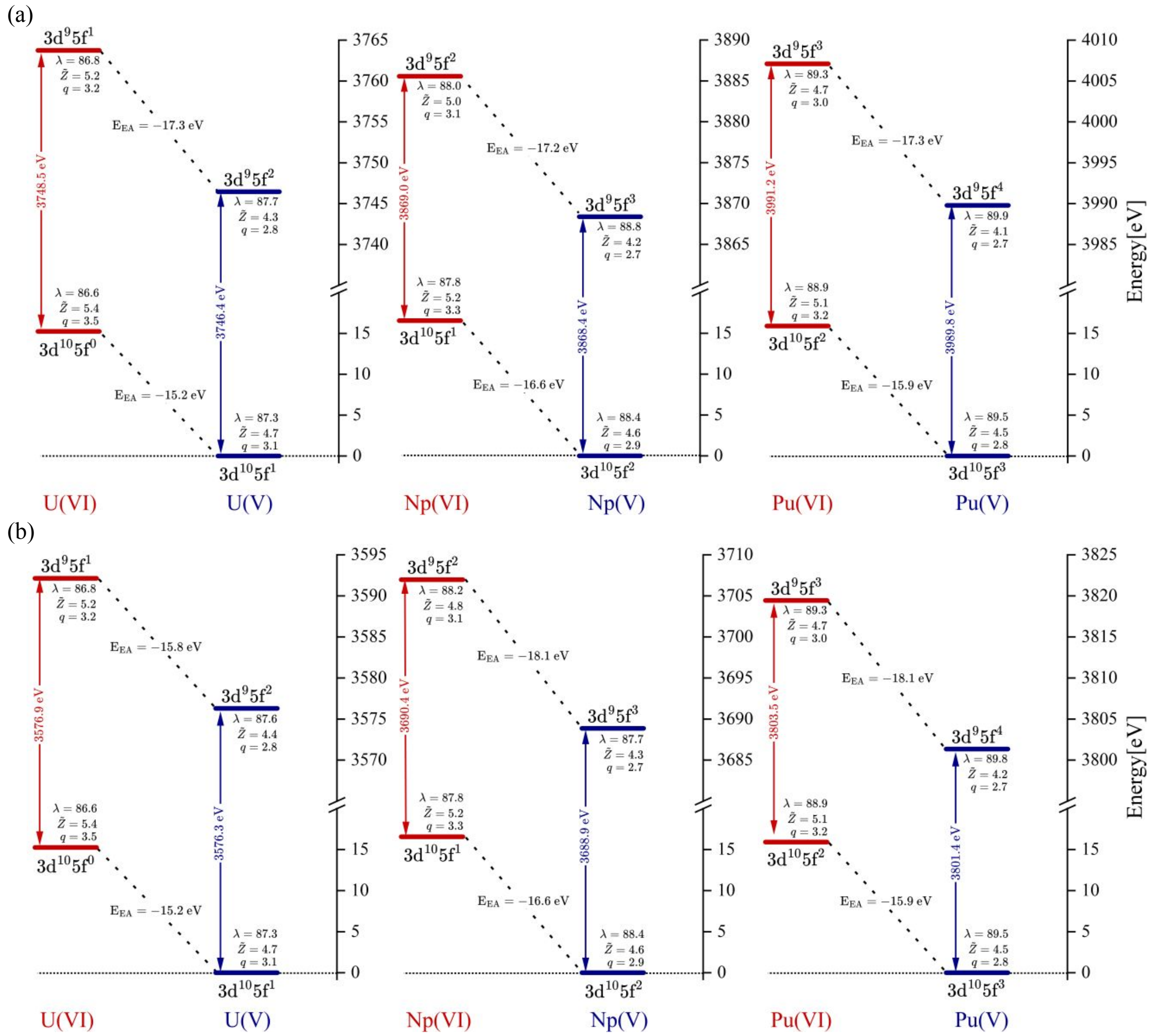

Figure S7. Complete set of RAS(SD) (a) M_4-edge and (b) M_5-edge state energy diagrams. Details on the quantities plotted are the same as those for the figure presented in the main manuscript (figure 4).

Detailed Assignments

Table S9. Assignments for RAS(SD) $[An(VI)O_2]^{2+}$ Systems.

System	Transition	State	Energy (eV)	3d	π_u	σ_u	ϕ_u	δ_u	π_u*	σ_u*
$[UO_2]^{2+}$ M_4-Edge	-	GS	0	10.00	3.92	1.95	0.00	0.01	0.08	0.03
	1.1	2286/2287	3748.5	9.00	3.95	1.97	0.69	0.29	0.09	0.01
	2.2	2323	3750.6	9.00	3.92	1.68	0.22	0.42	0.73	0.02
	3.2	2899	3755.1	9.00	3.41	1.71	0.38	0.57	0.78	0.16
$[UO_2]^{2+}$ M_5-Edge	-	GS	0	10.00	3.92	1.95	0.00	0.01	0.08	0.03
	1.1	48/49	3576.9	9.00	3.97	1.98	0.56	0.32	0.17	0.01
	2.3	153	3578.8	9.00	3.86	1.64	0.26	0.61	0.62	0.02
	3.1	1288	3585.3	9.00	3.61	1.62	0.12	0.25	1.02	0.38
$[NpO_2]^{2+}$ M_4-Edge	-	GS	0	10.00	3.91	1.95	0.90	0.12	0.09	0.03
	1.3	8481/8482	3869	9.00	3.94	1.96	0.91	1.05	0.11	0.02
	2.4	8596/8595	3871.2	9.00	3.74	1.84	0.93	0.71	0.67	0.11
	3.4	10372	3875.7	9.00	3.20	1.83	1.21	0.96	0.76	0.05
$[NpO_2]^{2+}$ M_5-Edge	-	GS	0	10.00	3.91	1.95	0.90	0.12	0.09	0.03
	1.2	95/96	3688.3	9.00	3.95	1.98	0.77	1.10	0.19	0.01
	1.6	329/330	3690.4	9.00	3.85	1.85	0.89	0.85	0.55	0.02
	2.1	4312	3696	9.00	3.35	1.71	1.00	0.76	1.08	0.10
$[PuO_2]^{2+}$ M_4-Edge	-	GS	0	10.00	3.88	1.95	0.97	1.03	0.13	0.04
	1.2	13028	3986.2	9.00	3.39	1.66	1.38	1.48	1.05	0.03
	2.1	13187	3991.2	9.00	3.70	1.86	1.18	1.67	0.57	0.03
	2.7	13420	3993.4	9.00	3.70	1.87	1.28	1.24	0.88	0.03
$[PuO_2]^{2+}$ M_5-Edge	-	GS	0	10.00	3.88	1.95	0.97	1.03	0.13	0.04
	1.1	117/118	3801.8	9.00	3.94	1.98	1.35	1.38	0.35	0.01
	1.5	401	3803.5	9.00	3.90	1.96	1.22	1.13	0.78	0.01
	2.1	6318	3809.3	9.00	3.32	1.74	1.43	1.35	1.08	0.08

Table S10. Assignments for RAS(SD) $[An(V)O_2]^+$ Systems.

System	Transition	State	Energy	3d	πu	σu	ϕu	δu	πu*	σu*
$[UO_2]^+$ M_4-Edge	-	GS	0	10.00	3.95	1.95	0.92	0.09	0.05	0.04
	1.4	6784/6783	3746.4	9.00	3.70	1.88	0.95	1.04	0.35	0.07
	2.4	6944/6943	3748.1	9.00	3.80	1.85	0.86	0.69	0.74	0.06
	3.6	7805/7806	3751.9	9.00	3.63	1.47	1.05	0.98	0.73	0.14
$[UO_2]^+$ M_5-Edge	-	GS	0	10.00	3.95	1.95	0.92	0.09	0.05	0.04
	1.1	96/95	3574.5	9.00	3.96	1.96	0.77	1.04	0.24	0.03
	1.6	270/269	3576.3	9.00	3.96	1.94	1.18	0.48	0.41	0.04
	2.7	1880/1879	3580.7	9.00	3.49	1.57	0.99	1.04	0.81	0.10
$[NpO_2]^+$ M_4-Edge	-	GS	0	10.00	3.93	1.94	0.97	1.02	0.09	0.05
	1.1	12304	3859.7	9.00	3.44	1.72	1.22	1.44	0.94	0.25
	1.2	12653	3867.2	9.00	3.88	1.91	1.06	1.74	0.35	0.05
	1.6	12814	3868.4	9.00	3.71	1.81	1.26	1.32	0.82	0.08
	1.12	13453	3870.8	9.00	3.75	1.70	1.25	1.17	1.04	0.09
$[NpO_2]^+$ M_5-Edge	-	GS	0	10.00	3.93	1.94	0.97	1.02	0.09	0.05
	1.1	117/118	3686.7	9.00	3.96	1.95	1.32	1.18	0.55	0.04
	1.4	383/384	3688.2	9.00	3.95	1.94	1.37	1.13	0.57	0.04
	1.6	529	3688.9	9.00	3.94	1.92	1.18	1.03	0.88	0.05
	2.5	2420	3692.3	9.00	3.67	1.48	1.24	1.57	0.92	0.12
	2.9	3003	3692.9	9.00	3.63	1.45	1.31	1.49	1.01	0.11
$[PuO_2]^+$ M_4-Edge	-	GS	0	10.00	3.92	1.93	1.09	1.87	0.12	0.06
	1.4	17579/17580	3989.8	9.00	3.66	1.77	1.50	1.76	1.19	0.12
	1.6	17701	3990.3	9.00	3.71	1.76	1.52	1.69	1.22	0.10
	1.12	18290/18289	3992.0	9.00	3.76	1.77	1.57	1.71	1.10	0.09
	1.14	18814	3993.0	9.00	3.75	1.81	1.58	1.58	1.13	0.16
$[PuO_2]^+$ M_5-Edge	-	GS	0	10.00	3.92	1.93	1.09	1.87	0.12	0.06
	1.1	97/98	3799.2	9.00	3.95	1.95	1.54	1.80	0.73	0.04
	1.8	623/624	3801.4	9.00	3.95	1.93	1.56	1.51	1.01	0.04
	2.2	3381/3382	3805.3	9.00	3.72	1.52	1.54	1.72	1.34	0.16

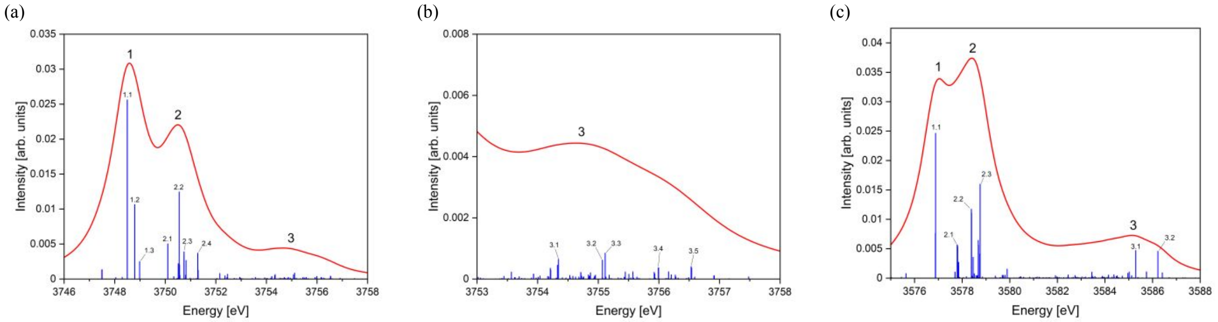

Figure S8. (a,b) U M_4-edge and XANES for $[U(VI)O_2]^{2+}$ and (c) U M_5-edge XANES for $[U(VI)O_2]^{2+}$.

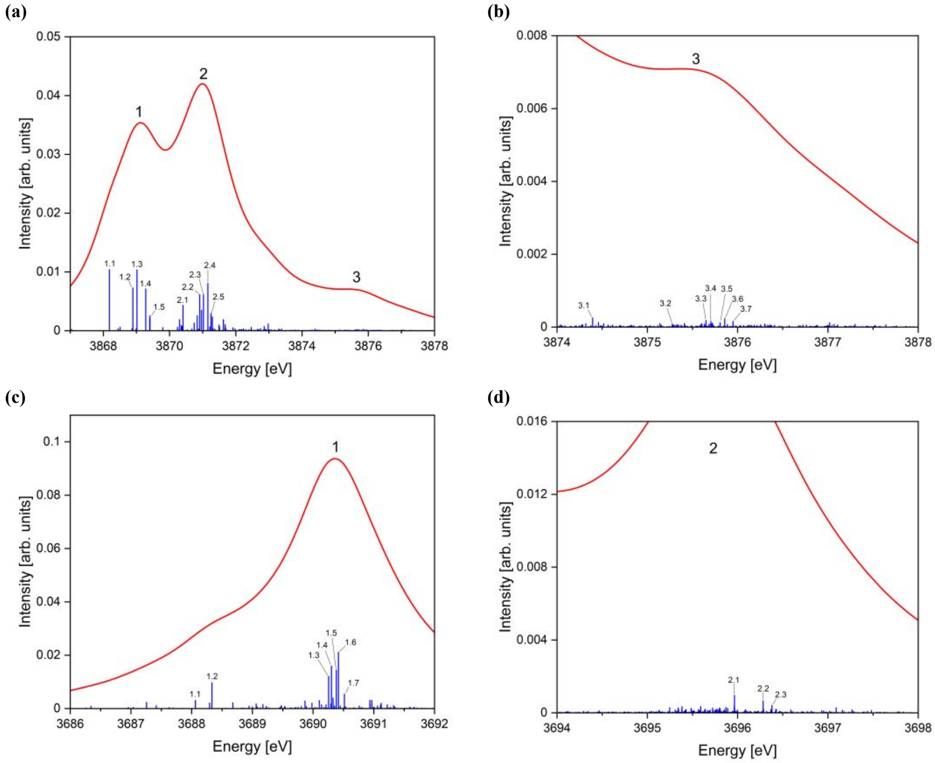

Figure S9. (a,b) Np M_4-edge and (c,d) M_5-edge XANES for $[NpO_2]^{2+}$.

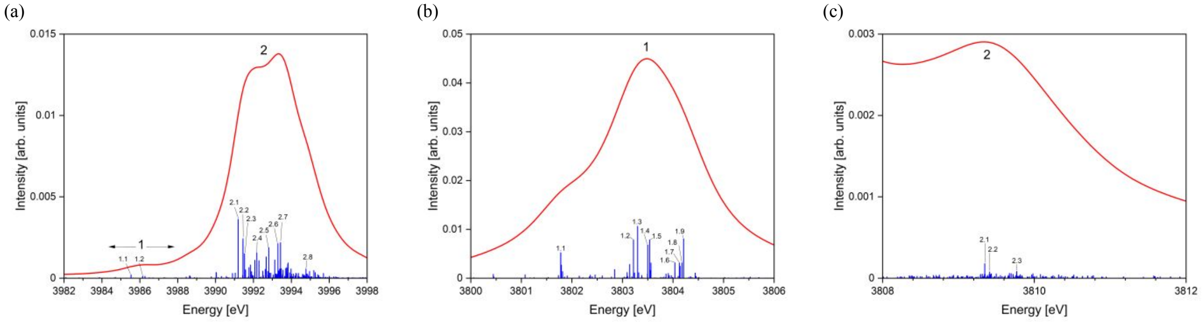

Figure S10. (a) Pu M_4-edge XANES for $[Pu(VI)O_2]^{2+}$ and (b,c) Pu M_5-edge XANES for $[Pu(VI)O_2]^{2+}$.

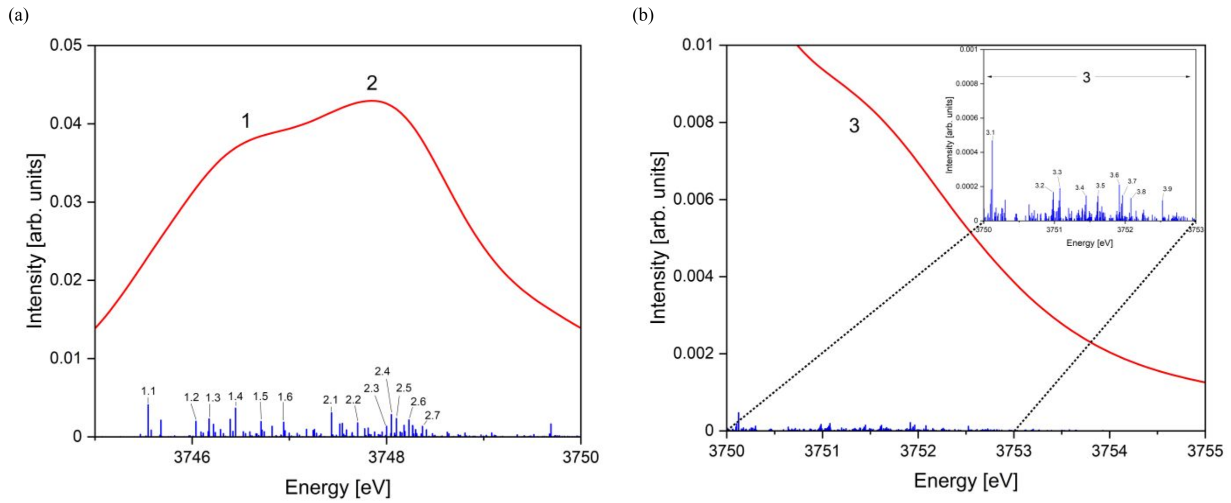

Figure S11. (a,b) U M_4-edge XANES for $[U(V)O_2]^+$.

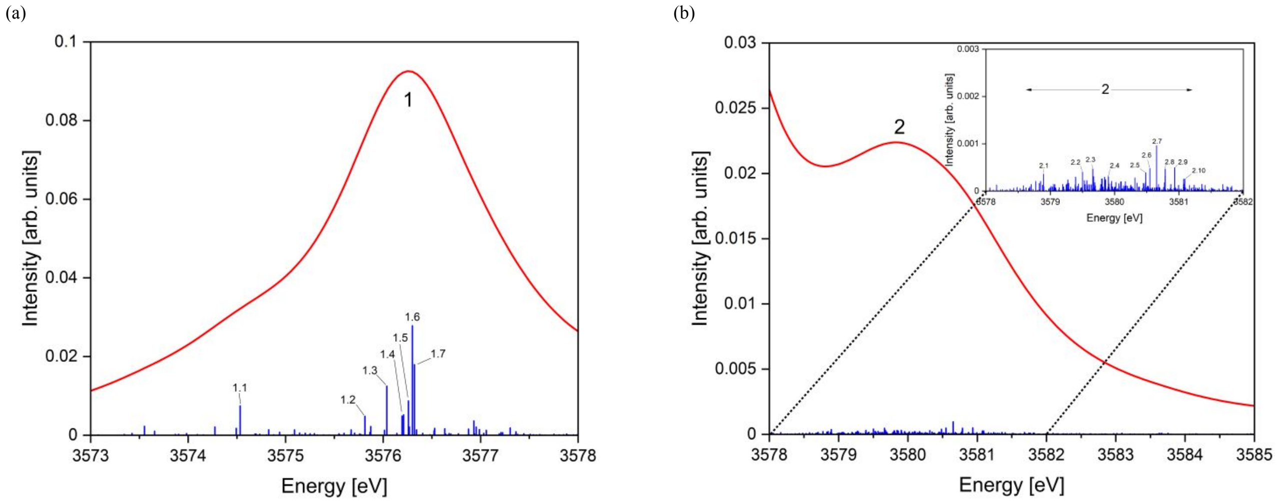

Figure S12. (a,b) U M_5-edge XANES for $[U(V)O_2]^+$.

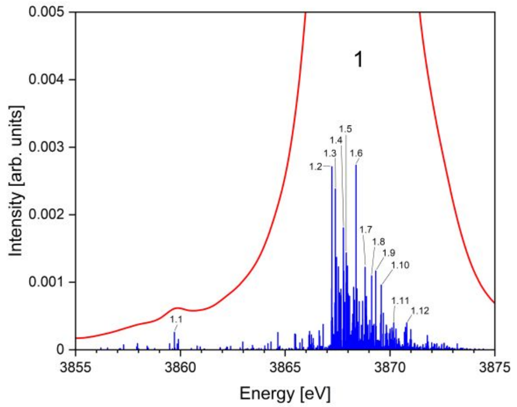

Figure S13. Np M_4-edge XANES for $[Np(V)O_2]^+$.

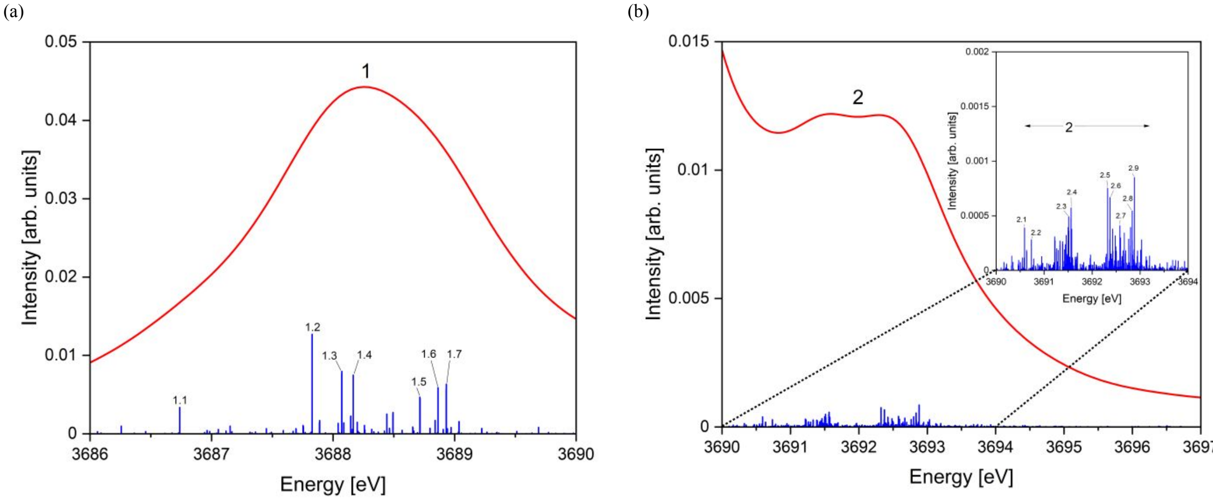

Figure S14. (a,b) Np M_5-edge XANES for $[Np(V)O_2]^+$.

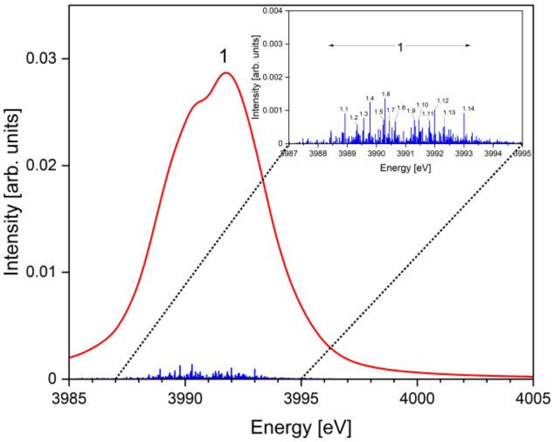

Figure S15. Pu M_4-edge XANES for $[Pu(V)O_2]^+$.

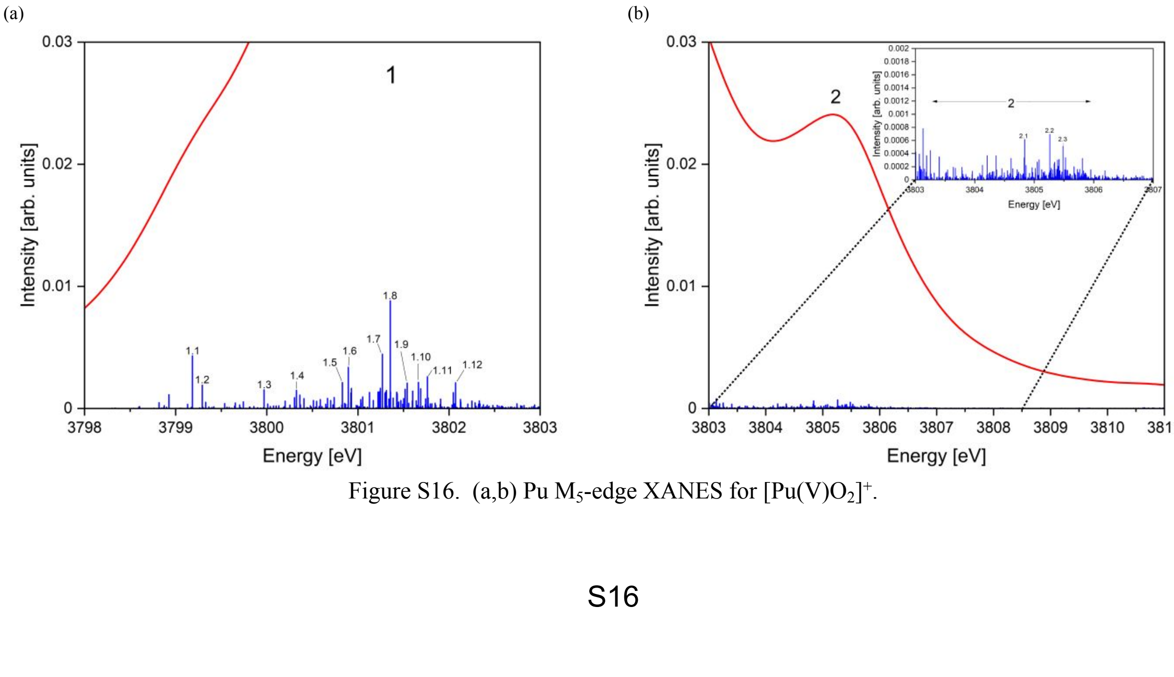

Figure S16. (a,b) Pu M_5-edge XANES for $[Pu(V)O_2]^+$.

Peak Position Measurements:

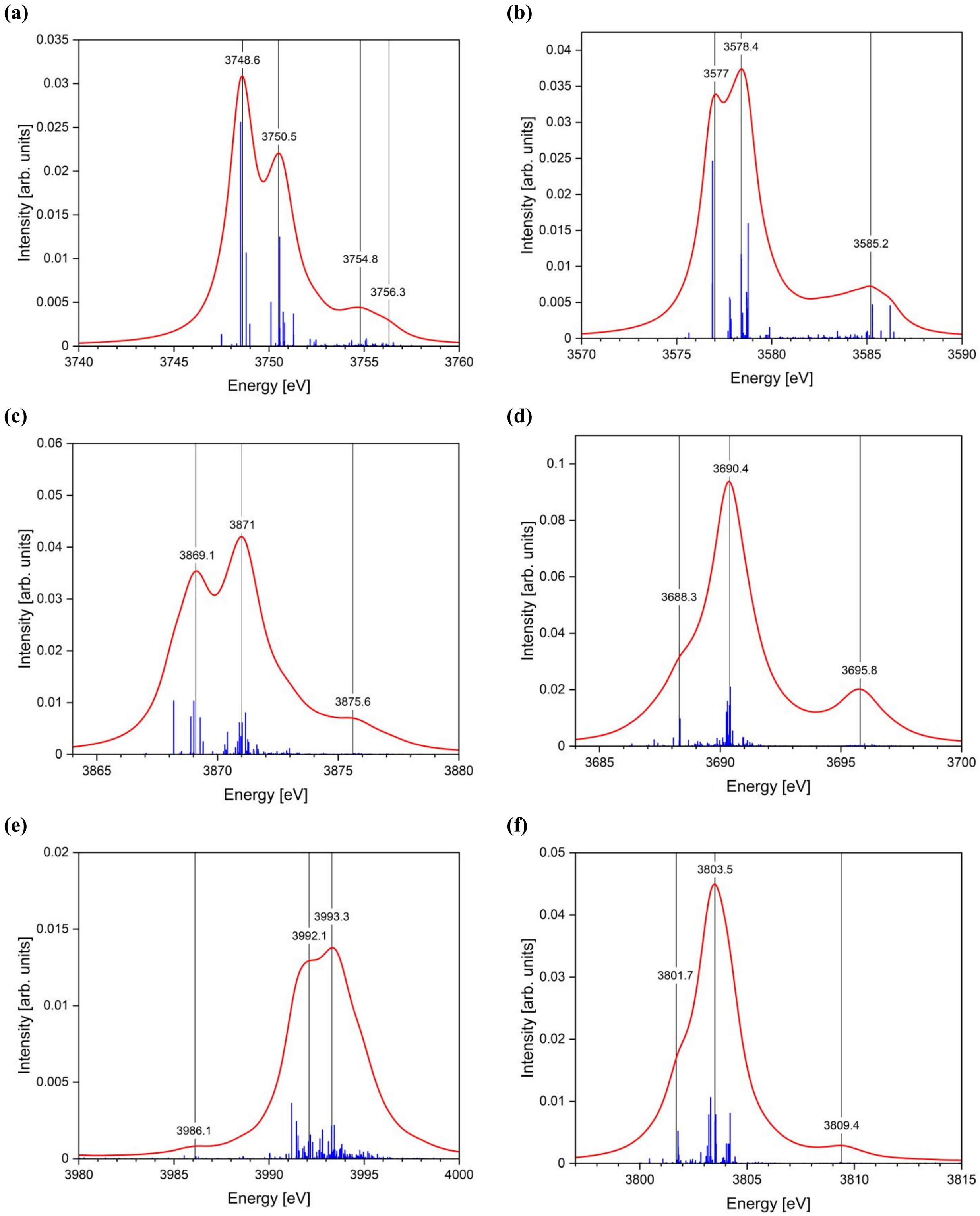

Figure S17. Peak energy position measurements for An(VI) systems. (a) U M_4-edge and (b) M_5-edge XANES of $[UO_2]^{2+}$. (c) Np M_4-edge and (d) M_5-edge XANES of $[NpO_2]^{2+}$. (e) Pu M_4-edge and (f) M_5-edge XANES of $[PuO_2]^{2+}$.

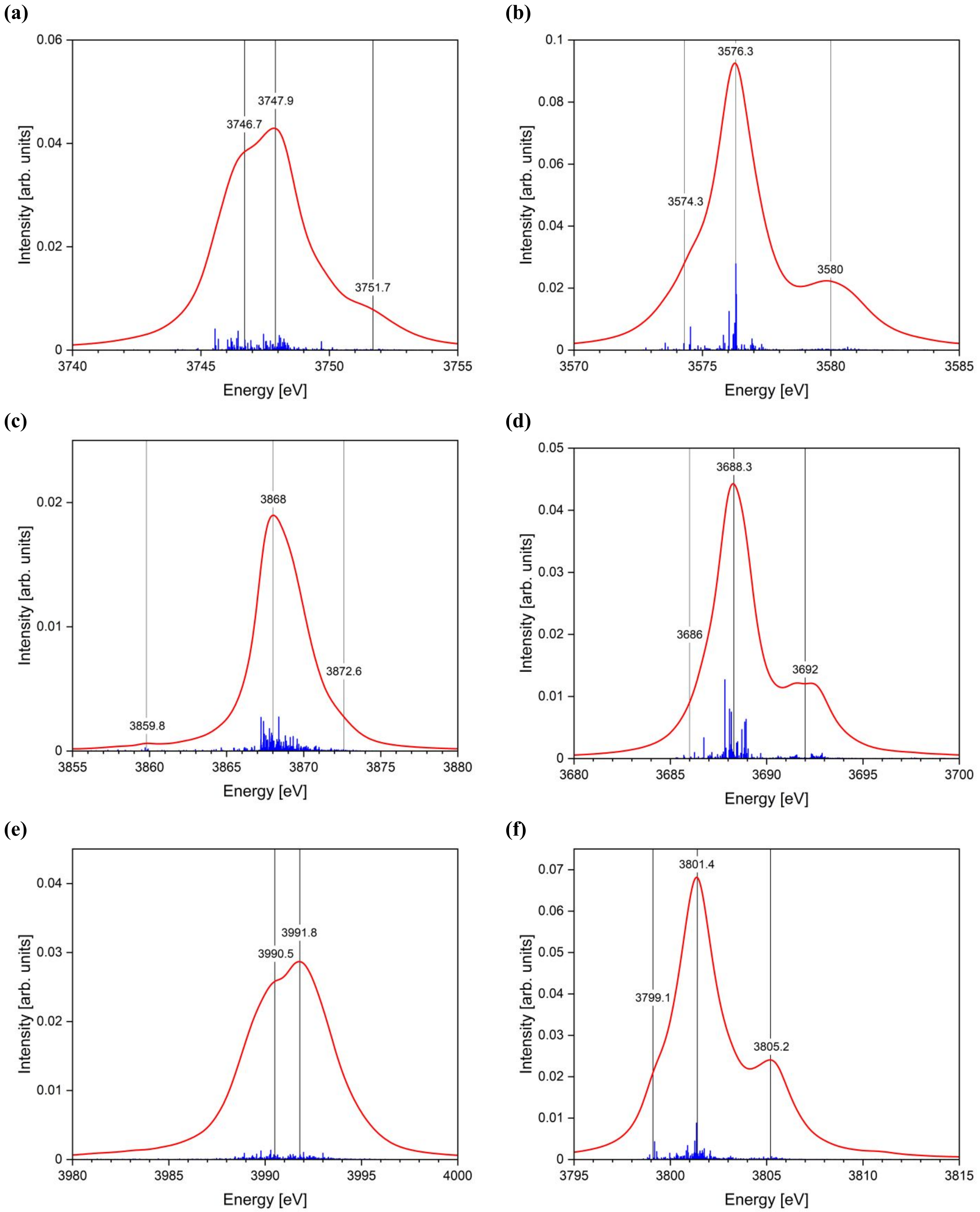

Figure S18. Peak energy position measurements for An(V) systems. (a) U M_4-edge and (b) M_5-edge XANES of $[UO_2]^+$. (c) Np M_4-edge and (d) M_5-edge XANES of $[NpO_2]^+$. (e) Pu M_4-edge and (f) M_5-edge XANES of $[PuO_2]^+$.

Pu M_5-edge XANES RAS(SD) simulation using experimental bond length:

$[PuO_2]^{2+}$ in D_{2h} symmetry was simulated using the RAS(SD) approach outlined in the computational details.

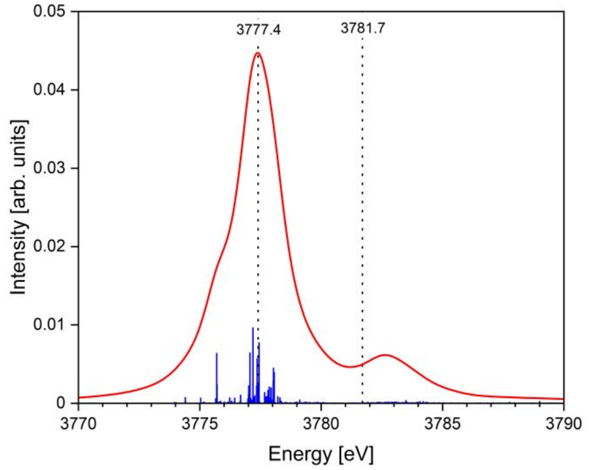

Figure S19. $[PuO_2]^{2+}$ M_5-edge RAS(SD) XANES at the experimental 1.74Å Pu-O bond length. Dashed lines show the experimental peak positions. Spectrum was shifted 26.1 eV to align the first peak with experiment, so the relative positions of simulation can be compared to experiment.

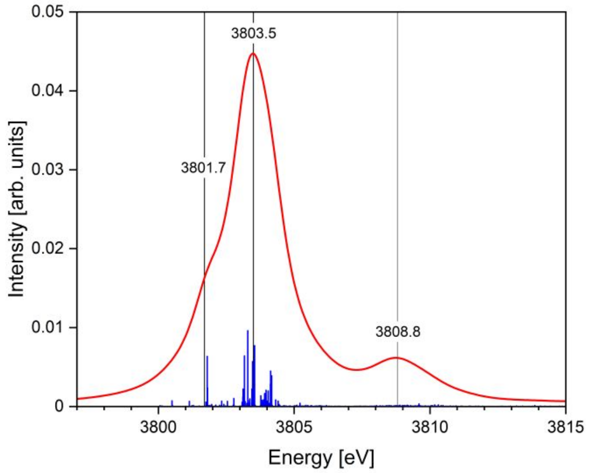

Figure S20. $[PuO_2]^{2+}$ M_5-edge RAS(SD) XANES at the experimental 1.74Å Pu-O bond length. Lines show the simulated peak positions.

Table of RAS(SD) Peak Positions:

Table S11. RAS(SD) simulated peak positions and energy shifts between the main absorption peak and the peak assigned to 5f-σ^* (σ^*-Shift, eV).

Simulation	Peak	Position (eV)	σ*-Shift
U(VI) M4 edge	1	3748.6	
	2	3750.5	
	3	3754.8	6.2
U(V) M4 edge	1	3746.7	
	2	3747.9	
	3	3751.7	5.0
U(VI) M5 edge	1	3577.0	
	2	3578.4	
	3	3585.2	8.2
U(V) M5 edge	Shoulder	3574.3	
	1	3576.3	
	2	3580.0	3.7
Np(VI) M5 edge	Shoulder	3688.3	
	1	3690.4	
	2	3695.8	5.4
Np(V) M5 edge	Shoulder	3686.0	
	1	3688.3	
	2	3692.0	3.7
Pu(VI) M5 edge	Shoulder	3801.7	
	1	3803.5	
	2	3809.4	5.9
Pu(V) M5 edge	Shoulder	3799.1	
	1	3801.4	
	2	3805.2	3.8

Table S12. RAS(SD) simulation peak positions and energy shifts between the main absorption peak and the peak assigned to 5f-σ^*. Simulations performed using experimental bond lengths of 1.76, 1.75 and 1.74 Å for U, Np and Pu, respectively. U and Np results are taken from a prior study[19] while Pu data is taken from peaks measured in figure S20.

Simulation	Peak	Position (eV)	σ*-Shift
U(VI) M4 edge:	1	3748.6	
	2	3750.5	
	3	3754.7	6.1
Np(VI) M5 edge:	Shoulder	3688.5	
	1	3690.4	
	2	3695.3	4.9
Pu(VI) M5 edge:	Shoulder	3801.7	
	1	3803.5	
	2	3808.8	5.3

RAS(SD) QTAIM Data

Table S13. Table of QTAIM data for the GS and CESs identified as belonging to key transitions in the RAS(SD) XANES simulations. QTAIM differences (Δ) are with respect to the GS.

Simulation	Peak	Transition	Assignment	ρ(An-O)	δ(An-O)	λ(An)	λ(O)	Δρ(An-O)	Δδ(An-O)	Δλ(An)	Δλ(O)
U(VI) M4 edge:			GS	0.35	1.86	86.64	7.79				
	1	1.1	3d→5f-ϕ/δ	0.35	2.01	86.83	7.52	0.00	0.15	0.20	-0.26
	2	2.2	3d→5f-π*	0.34	1.68	87.02	7.76	-0.01	-0.18	0.38	-0.02
	3	3.2	3d→5f-σ*	0.33	1.48	87.25	7.87	-0.02	-0.38	0.62	0.08
Np(VI) M5 edge:			GS	0.37	1.89	87.76	7.69				
	Shldr.	1.2	3d→5f-ϕ/δ/π*	0.37	1.92	88.01	7.51	0.00	0.03	0.25	-0.18
	1	1.6	3d→5f-ϕ/δ/π*	0.36	1.74	88.15	7.63	-0.01	-0.15	0.39	-0.06
	2	2.1	3d→5f-σ*	0.35	1.47	88.32	7.84	-0.02	-0.42	0.56	0.15
Pu(VI) M5 edge:			GS	0.38	1.90	88.89	7.62				
	Shdr.	1.1	3d→5f-ϕ/δ/π*	0.38	1.86	89.18	7.48	0.00	-0.04	0.30	-0.14
	1	1.5	3d→5f-ϕ/δ/π*	0.38	1.74	89.27	7.56	0.00	-0.16	0.39	-0.06
	2	2.1	3d→5f-σ*	0.37	1.49	89.40	7.77	-0.01	-0.41	0.52	0.15
U(V) M4 edge:			GS	0.29	1.65	87.30	8.18				
	1	1.4	3d→5f-ϕ/δ/π*	0.28	1.52	87.70	8.11	-0.01	-0.14	0.41	-0.07
	2	2.4	3d→5f-ϕ/δ/π*	0.28	1.47	87.70	8.15	-0.01	-0.18	0.41	-0.03
	3	3.6	3d→5f-σ*	0.26	1.30	87.84	8.26	-0.03	-0.35	0.55	0.08
Np(V) M5 edge:			GS	0.30	1.67	88.41	8.10				
	Shldr.	1.1	3d→5f-ϕ/δ/π*	0.30	1.59	88.69	8.03	0.00	-0.08	0.28	-0.07
	1	1.6	3d→5f-ϕ/δ/π*	0.30	1.53	88.74	8.06	0.00	-0.14	0.32	-0.04
	2	2.5	3d→5f-σ*	0.28	1.33	88.87	8.21	-0.02	-0.34	0.46	0.11
Pu(V) M5 edge:			GS	0.31	1.68	89.53	8.03				
	Shldr.	1.1	3d→5f-ϕ/δ/π*	0.31	1.57	89.79	7.99	0.00	-0.11	0.26	-0.05
	1	1.8	3d→5f-ϕ/δ/π*	0.31	1.53	89.80	8.02	0.00	-0.14	0.27	-0.02
	2	2.2	3d→5f-σ*	0.29	1.33	89.92	8.18	-0.02	-0.35	0.39	0.15
U(VI) M5 edge:			GS	0.35	1.86	86.64	7.79				
	1	1.1	3d→5f-ϕ/δ/π*	0.35	1.95	86.83	7.57	0.00	0.09	0.20	-0.21
	2	2.3	3d→5f-ϕ/δ/π*	0.33	1.68	87.02	7.76	-0.02	-0.18	0.38	-0.03
	3	3.1	3d→5f-σ*	0.34	1.42	87.19	7.96	-0.01	-0.44	0.55	0.18
U(V) M5 edge:			GS	0.29	1.65	87.30	8.18				
	Shldr.	1.1	3d→5f-ϕ/δ/π*	0.29	1.66	87.53	8.04	0.00	0.01	0.23	-0.14
	1	1.6	3d→5f-ϕ/δ/π*	0.29	1.59	87.59	8.08	0.00	-0.06	0.30	-0.10
	2	2.7	3d→5f-σ*	0.26	1.31	87.87	8.24	-0.03	-0.34	0.57	0.06

Table S14. Table of QTAIM data for the GS and CESs identified as belonging to key transitions in the RAS(SD) XANES simulations performed using actinyl systems set to experimental bond lengths. QTAIM differences (Δ) are with respect to the GS. U and Np data taken from the ESI of our previous study and presented here for convenience. [19] Pu data is reported for the RAS(SD) simulations performed for this study and correspond to states attributed to transitions in figure S20.

Simulation	Peak	Transition	Assignment	ρ(An-O)	δ(An-O)	λ(An)	λ(O)	Δρ(An-O)	Δδ(An-O)	Δλ(An)	Δλ(O)
U(VI) M_4 edge: (Previous Study)			GS	0.33	1.85	86.67	7.78				
	1	1.1	3d→5f-ϕ/δ	0.34	2.03	86.79	7.51	0.01	0.18	0.12	-0.27
	2	2.4	3d→5f-π*	0.32	1.65	87.05	7.77	-0.01	-0.20	0.38	-0.01
	3	3.11	3d→5f-σ*	0.32	1.48	87.28	7.85	-0.01	-0.37	0.61	0.07
Np(VI) M_5 edge: (Previous Study)			GS	0.34	1.86	87.83	7.69				
	1	1.8	3d→5f-ϕ/δ/π*	0.34	1.72	88.21	7.61	0.00	-0.14	0.38	-0.08
	2	2.5	3d→5f-σ*	0.33	1.44	88.37	7.85	-0.01	-0.42	0.54	0.16
Pu(VI) M_5 edge: (Current Study)			GS	0.34	1.85	88.97	7.63				
	Shldr	1.1	3d→5f-ϕ/δ/π*	0.35	1.79	89.28	7.49	0.00	-0.06	0.31	-0.13
	1	1.5	3d→5f-ϕ/δ/π*	0.35	1.72	89.34	7.55	0.00	-0.13	0.37	-0.08
	2	2.5	3d→5f-σ*	0.33	1.46	89.42	7.78	-0.01	-0.38	0.45	0.15

5. RAS(S) Results

An M4/5-edge XANES spectra:

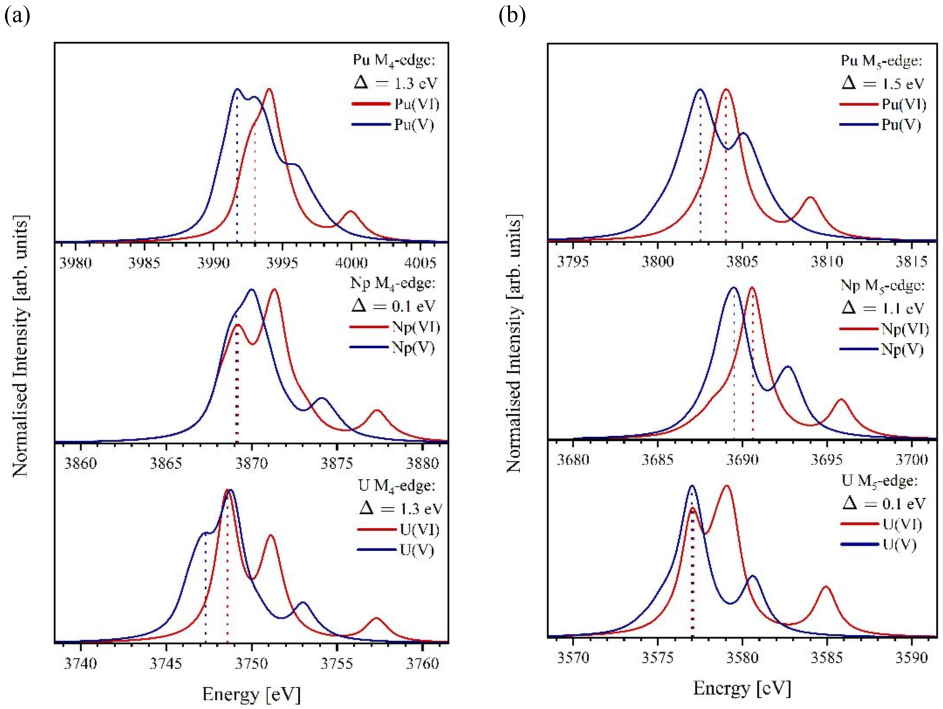

Figure S21. RAS(S) simulated XANES spectra for actinyls in +6 and +5 oxidation state.

Table S15. RAS(S) simulation peak energy positions			
Simulation	Peak	Position	Δ(ϕ/δ/π* - σ*)
U(VI) M4 edge:	1	3748.6	
	2	3751.2	
	3	3757.3	8.7
U(V) M4 edge:	1	3747.3	
	2	3748.8	
	3	3753.1	5.8
U(VI) M5 edge:	1	3577.1	
	2	3579.1	
	3	3584.9	7.8
U(V) M5 edge:	Shoulder		
	1	3577.1	
	2	3580.7	3.6
Np(VI) M5 edge:	Shoulder		
	1	3690.6	
	2	3695.9	5.3
Np(V) M5 edge:	Shoulder		
	1	3689.6	
	2	3692.7	3.1
Pu(VI) M5 edge:	Shoulder		
	1	3804.1	
	2	3809.1	5.0
Pu(V) M5 edge:	Shoulder		
	1	3802.6	
	2	3805.1	2.5

Overall XANES Assignments

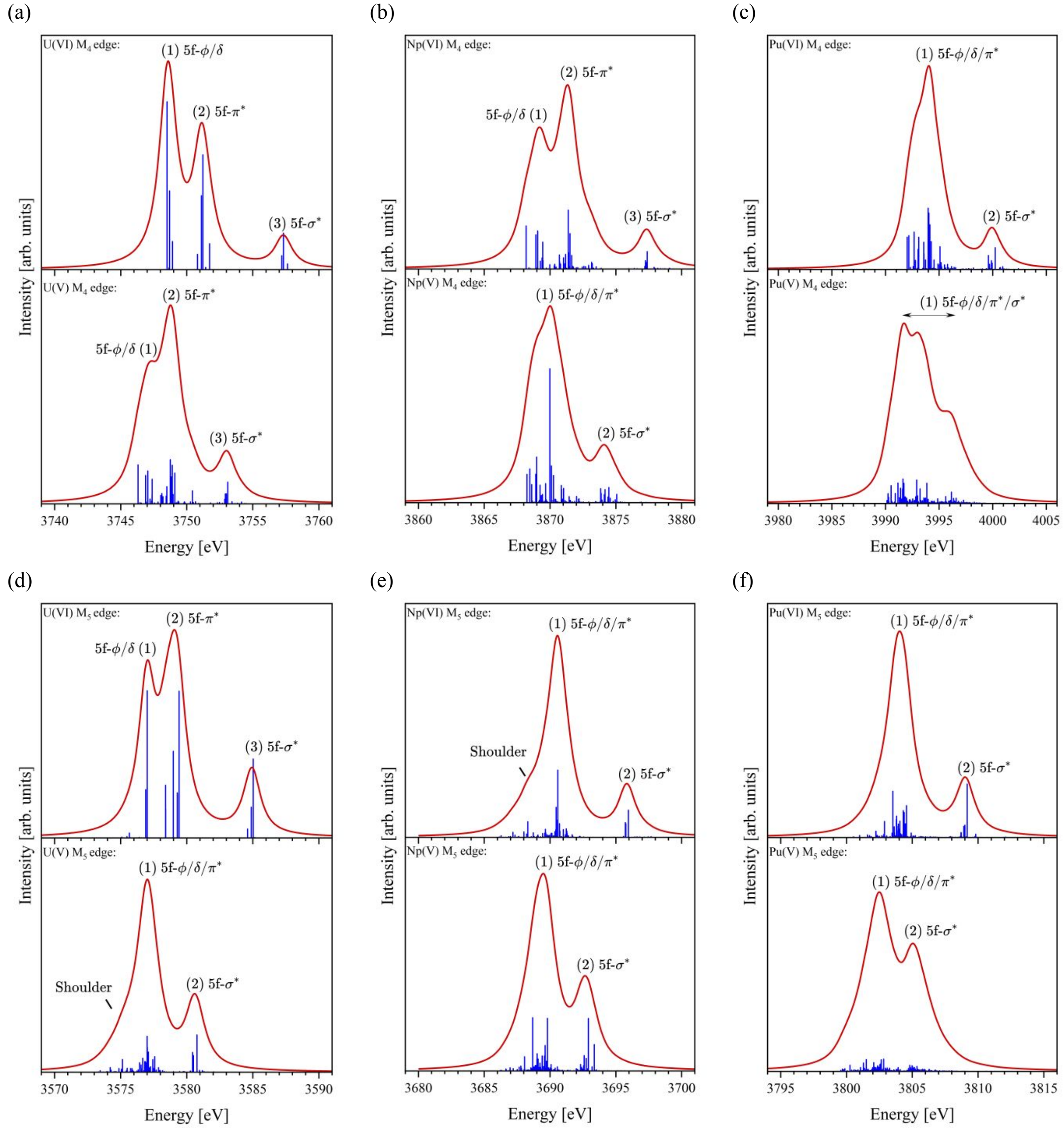

Figure S22. Overall RAS(S) XANES assignments.

State-Energy Diagrams

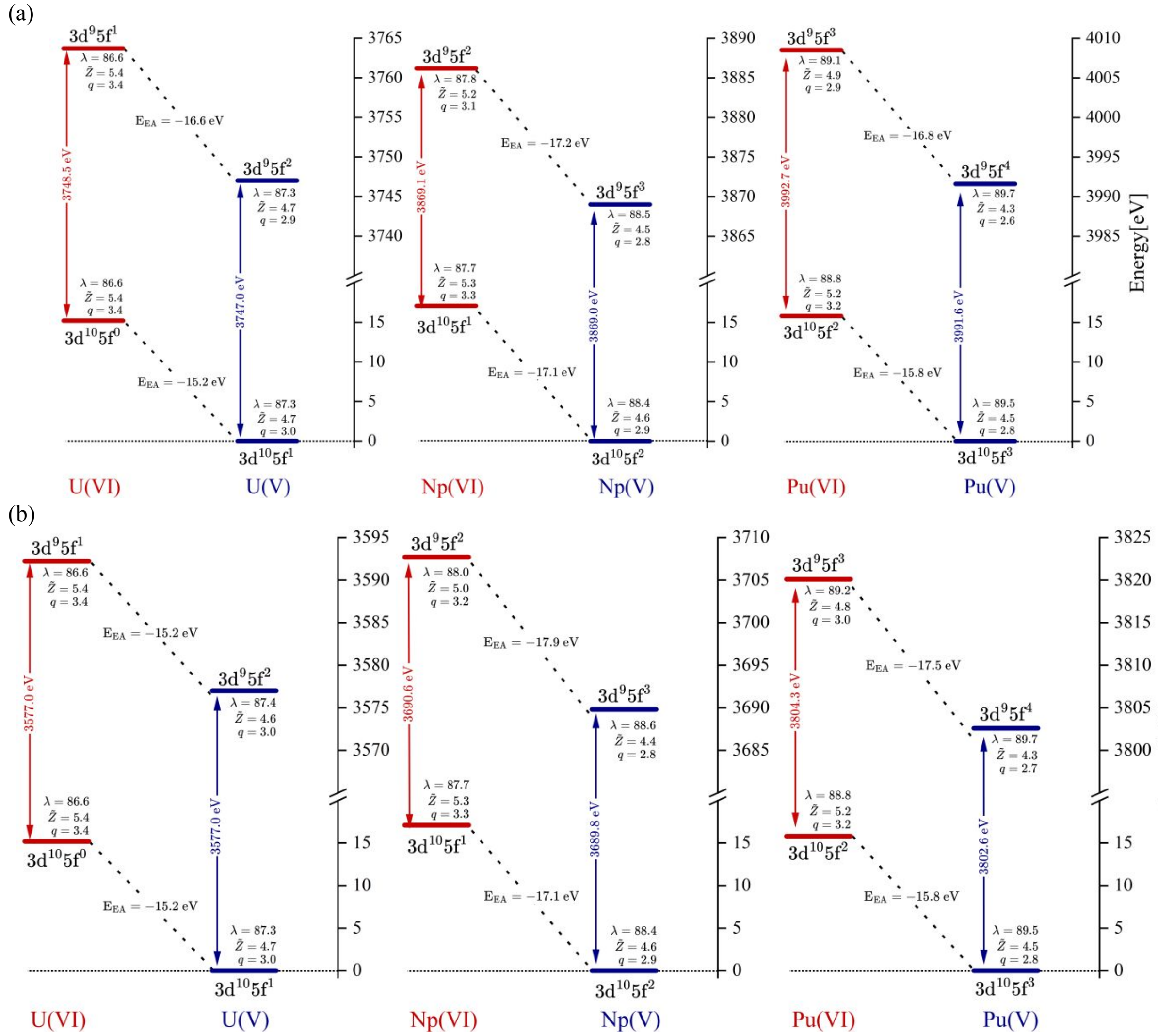

Figure S23. Complete set of RAS(S) (a) M_4-edge and (b) M_5-edge state energy diagrams. Details on the quantities plotted are the same as those for the figure presented in the main manuscript (figure 4).

Additional Author Information:

Kurtis Stanistreet-Welsh:
Present Address:
[#] Department of Chemistry, University at Buffalo State University of New York, Buffalo, New York 14260-3000, United States

References:

(1) Balasubramani, S. G.; Chen, G. P.; Coriani, S.; Diedenhofen, M.; Frank, M. S.; Franzke, Y. J.; Furche, F.; Grotjahn, R.; Harding, M. E.; Hättig, C.; Hellweg, A.; Helmich-Paris, B.; Holzer, C.; Huniar, U.; Kaupp, M.; Marefat Khah, A.; Karbalaei Khani, S.; Müller, T.; Mack, F.; Nguyen, B. D.; Parker, S. M.; Perlt, E.; Rappoport, D.; Reiter, K.; Roy, S.; Rückert, M.; Schmitz, G.; Sierka, M.; Tapavicza, E.; Tew, D. P.; van Wüllen, C.; Voora, V. K.; Weigend, F.; Wodyński, A.; Yu, J. M. TURBOMOLE: Modular program suite for ab initio quantum-chemical and condensed-matter simulations. *The Journal of Chemical Physics* **2020**, *152*, 184107.

(2) Adamo, C.; Barone, V. Toward reliable density functional methods without adjustable parameters: The PBE0 model. *The Journal of Chemical Physics* **1999**, *110*, 6158–6170.

(3) Ernzerhof, M.; Scuseria, G. E. Assessment of the Perdew–Burke–Ernzerhof exchange-correlation functional. *The Journal of Chemical Physics* **1999**, *110*, 5029–5036.

(4) Weigend, F.; Ahlrichs, R. Balanced basis sets of split valence, triple zeta valence and quadruple zeta valence quality for H to Rn: Design and assessment of accuracy. *Phys. Chem. Chem. Phys.* **2005**, *7*, 3297–3305.

(5) Küchle, W.; Dolg, M.; Stoll, H.; Preuss, H. Energy-adjusted pseudopotentials for the actinides. Parameter sets and test calculations for thorium and thorium monoxide. *The Journal of Chemical Physics* **1994**, *100*, 7535–7542.

(6) Klamt, A.; Schüürmann, G. COSMO: a new approach to dielectric screening in solvents with explicit expressions for the screening energy and its gradient. *J. Chem. Soc., Perkin Trans.* **1993**, *2*, 799–805.

(7) Tarlton, M. L.; Fajen, O. J.; Kelley, S. P.; Kerridge, A.; Malcomson, T.; Morrison, T. L.; Shores, M. P.; Xhani, X.; Walensky, J. R. Systematic Investigation of the Molecular and Electronic Structure of Thorium and Uranium Phosphorus and Arsenic Complexes. *Inorganic Chemistry* **2021**, *60*, 10614–10630.

(8) Behrle, A. C.; Myers, A. J.; Kerridge, A.; Walensky, J. R. Coordination Chemistry and QTAIM Analysis of Homoleptic Dithiocarbamate Complexes, $M(S_2CN^iPr_2)_4$ (M = Ti, Zr, Hf, Th, U, Np). *Inorganic Chemistry* **2018**, *57*, 10518–10524.

(9) Behrle, A. C.; Kerridge, A.; Walensky, J. R. Dithio- and Diselenophosphinate Thorium(IV) and Uranium(IV) Complexes: Molecular and Electronic Structures, Spectroscopy, and Transmetalation Reactivity. *Inorganic Chemistry* **2015**, *54*, 11625–11636.

(10) Hay, P. J.; Martin, R. L.; Schreckenbach, G. Theoretical Studies of the Properties and Solution Chemistry of AnO_2^{2+} and AnO_2^{+} Aquo Complexes for An = U, Np, and Pu. *The Journal of Physical Chemistry A* **2000**, *104*, 6259–6270.

(11) Shamov, G. A.; Schreckenbach, G. Density Functional Studies of Actinyl Aquo Complexes Studied Using Small-Core Effective Core Potentials and a Scalar Four-Component Relativistic Method. *The Journal of Physical Chemistry A* **2005**, *109*, 10961–10974.

(12) Combes, J. M.; Chisholm-Brause, C. J.; Brown, G. E. J.; Parks, G. A.; Conradson, S. D.; Eller, P. G.; Triay, I. R.; Hobart, D. E.; Miejer, A. EXAFS spectroscopic study of neptunium(V) sorption at the .alpha.-iron hydroxide oxide (.alpha.-FeOOH)/water interface. Environmental Science & Technology 1992, 26, 376–382.

(13) Conradson, S. D. Application of X-Ray Absorption Fine Structure Spectroscopy to Materials and Environmental Science. *Applied Spectroscopy* **1998**, *52*, 252A–279A.

(14) Allen, P. G.; Bucher, J. J.; Shuh, D. K.; Edelstein, N. M.; Reich, T. Investigation of Aquo and Chloro Complexes of UO_2^{2+}, NpO_2^{+}, Np^{4+}, and Pu^{3+} by X-ray Absorption Fine Structure Spectroscopy. *Inorganic Chemistry* **1997**, *36*, 4676–4683.

(15) Malmqvist, P. A.; Rendell, A.; Roos, B. O. The restricted active space self-consistent-field method, implemented with a split graph unitary group approach. *The Journal of Physical Chemistry* **1990**, *94*, 5477–5482.

(16) Olsen, J.; Roos, B. O.; Jo/rgensen, P.; Jensen, H. J. A. Determinant based configuration interaction algorithms for complete and restricted configuration interaction spaces. *The Journal of Chemical Physics* **1988**, *89*, 2185–2192.

(17) Aquilante, F.; Autschbach, J.; Baiardi, A.; Battaglia, S.; Borin, V. A.; Chibotaru, L. F.; Conti, I.; De Vico, L.; Delcey, M.; Fdez. Galván, I.; Ferré, N.; Freitag, L.; Garavelli, M.; Gong, X.; Knecht, S.; Larsson, E. D.; Lindh, R.; Lundberg, M.; Malmqvist, P. .; Nenov, A.; Norell, J.; Odelius, M.; Olivucci, M.; Pedersen, T. B.; Pedraza-González, L.; Phung, Q. M.; Pierloot, K.; Reiher, M.; Schapiro, I.; Segarra-Martí, J.; Segatta, F.; Seijo, L.; Sen, S.; Sergentu, D.-C.; Stein, C. J.; Ungur, L.; Vacher, M.; Valentini, A.; Veryazov, V. Modern quantum chemistry with [Open]Molcas. *The Journal of Chemical Physics* **2020**, *152*, 214117.

(18) Aquilante, F.; Autschbach, J.; Carlson, R. K.; Chibotaru, L. F.; Delcey, M. G.; De Vico, L.; Fdez. Galván, I.; Ferré, N.; Frutos, L. M.; Gagliardi, L.; Garavelli, M.; Giussani, A.; Hoyer, C. E.; Li Manni, G.; Lischka, H.; Ma, D.; Malmqvist, P. .; Müller, T.; Nenov, A.; Olivucci, M.; Pedersen, T. B.; Peng, D.; Plasser, F.; Pritchard, B.; Reiher, M.; Rivalta, I.; Schapiro, I.; Segarra-Martí, J.; Stenrup, M.; Truhlar, D. G.; Ungur, L.; Valentini, A.; Vancoillie, S.; Veryazov, V.; Vysotskiy, V. P.; Weingart, O.; Zapata, F.; Lindh, R. Molcas 8: New capabilities for multiconfigurational quantum chemical calculations across the periodic table. *Journal of Computational Chemistry* **2016**, *37*, 506–541.

(19) Stanistreet-Welsh, K.; Kerridge, A. Bounding $[AnO_2]^{2+}$ (An = U, Np) covalency by simulated O K-edge and An M-edge X-ray absorption near-edge spectroscopy. *Phys. Chem. Chem. Phys.* **2023**, *25*, 23753–23760.

(20) Stanistreet-Welsh, K.; Kerridge, A. Quantifying Covalency and Environmental Effects in RASSCF-Simulated O K-Edge XANES of Uranyl. *Inorganic Chemistry* **2024**, *63*, 15115–15126.

(21) Roos, B. O.; Lindh, R.; Åke Malmqvist, P.; Veryazov, V.; Widmark, P.-O. New relativistic ANO basis sets for actinide atoms. *Chemical Physics Letters* **2005**, *409*, 295–299.

(22) Roos, B. O.; Lindh, R.; Malmqvist, P.-.; Veryazov, V.; Widmark, P.-O. Main Group Atoms and Dimers Studied with a New Relativistic ANO Basis Set. *The Journal of Physical Chemistry A* **2004**, *108*, 2851–2858.

(23) Wolf, A.; Reiher, M.; Hess, B. A. The generalized Douglas–Kroll transformation. *The Journal of Chemical Physics* **2002**, *117*, 9215–9226.

(24) Hess, B. A. Relativistic electronic-structure calculations employing a two-component no-pair formalism with external-field projection operators. *Phys. Rev. A* **1986**, *33*, 3742–3748.

(25) Hess, B. A. Applicability of the no-pair equation with free-particle projection operators to atomic and molecular structure calculations. *Phys. Rev. A* **1985**, *32*, 756–763.

(26) Douglas, M.; Kroll, N. M. Quantum electrodynamical corrections to the fine structure of helium. *Annals of Physics* **1974**, *82*, 89–155.

(27) Vancoillie, S.; Zhao, H.; Tran, V. T.; Hendrickx, M. F. A.; Pierloot, K. Multiconfigurational Second-Order Perturbation Theory Restricted Active Space (RASPT2) Studies on Mononuclear First-Row Transition-Metal Systems. *Journal of Chemical Theory and Computation* **2011**, *7*, 3961– 3977.

(28) Finley, J.; Åke Malmqvist, P.; Roos, B. O.; Serrano-Andrés, L. The multi-state CASPT2 method. *Chemical Physics Letters* **1998**, *288*, 299–306.

(29) Zobel, J. P.; Nogueira, J. J.; González, L. The IPEA dilemma in CASPT2. *Chem. Sci.* **2017**, *8*, 1482–1499.

(30) Forsberg, N.; Åke Malmqvist, P. Multiconfiguration perturbation theory with imaginary level shift. *Chemical Physics Letters* **1997**, *274*, 196–204.

(31) Sergentu, D.-C.; Duignan, T. J.; Autschbach, J. Ab Initio Study of Covalency in the Ground versus Core-Excited States and X-ray Absorption Spectra of Actinide Complexes. *The Journal of Physical Chemistry Letters* **2018**, *9*, 5583–5591.

(32) Ganguly, G.; Sergentu, D.-C.; Autschbach, J. Ab Initio Analysis of Metal–Ligand Bonding in An(COT)2 with An=Th, U in Their Ground- and Core-Excited States. *Chemistry – A European Journal* **2020**, *26*, 1776–1788.

(33) Qiao, Y.; Ganguly, G.; Booth, C. H.; Branson, J. A.; Ditter, A. S.; Lussier, D. J.; Moreau, L. M.; Russo, D. R.; Sergentu, D.-C.; Shuh, D. K.; Sun, T.; Autschbach, J.; Minasian, S. G. Enhanced 5f-δ bonding in $[U(C_7H_7)_2]^-$: C K-edge XAS, magnetism, and ab initio calculations. *Chem. Commun.* **2021**, *57*, 9562–9565.

(34) Sergentu, D.-C.; Autschbach, J. Covalency in actinide(iv) hexachlorides in relation to the chlorine K-edge X-ray absorption structure. *Chem. Sci.* **2022**, *13*, 3194–3207.

(35) Sergentu, D.-C.; Autschbach, J. X-ray absorption spectra of f-element complexes: insight from relativistic multiconfigurational wavefunction theory. *Dalton Trans.* **2022**, *51*, 1754–1764.

(36) Polly, R.; Schacherl, B.; Rothe, J.; Vitova, T. Relativistic Multiconfigurational Ab Initio Calculation of Uranyl 3d4f Resonant Inelastic X-ray Scattering. *Inorganic Chemistry* **2021**, *60*, 18764– 18776.

(37) Sauri, V.; Serrano-Andrés, L.; Shahi, A. R. M.; Gagliardi, L.; Vancoillie, S.; Pierloot, K. Multiconfigurational Second-Order Perturbation Theory Restricted Active Space (RASPT2) Method for Electronic Excited States: A Benchmark Study. *Journal of Chemical Theory and Computation* **2011**, *7*, 153–168.

(38) Hess, B. A.; Marian, C. M.; Wahlgren, U.; Gropen, O. A mean-field spin-orbit method applicable to correlated wavefunctions. *Chemical Physics Letters* **1996**, *251*, 365–371.

(39) Åke Malmqvist, P.; Roos, B. O.; Schimmelpfennig, B. The restricted active space (RAS) state interaction approach with spin–orbit coupling. *Chemical Physics Letters* **2002**, *357*, 230–240.

(40) Vitova, T.; Pidchenko, I.; Fellhauer, D.; Bagus, P. S.; Joly, Y.; Pruessmann, T.; Bahl, S.; GonzalezRobles, E.; Rothe, J.; Altmaier, M.; Denecke, M. A.; Geckeis, H. The role of the 5f valence orbitals of early actinides in chemical bonding. *Nature Communications* **2017**, *8*, 16053.

(41) Ehrman, J. N.; Shumilov, K.; Jenkins, A. J.; Kasper, J. M.; Vitova, T.; Batista, E. R.; Yang, P.; Li, X. Unveiling Hidden Shake-Up Features in the Uranyl M_4-Edge Spectrum. *JACS Au* **2024**, *4*, 1134–1141.

(42) Bagus, P. S.; Schacherl, B.; Vitova, T. Computational and Spectroscopic Tools for the Detection of Bond Covalency in Pu(IV) Materials. *Inorganic Chemistry* **2021**, *60*, 16090–16102.

(43) Kvashnina, K. O.; Butorin, S. M. High-energy resolution X-ray spectroscopy at actinide M4,5 and ligand K edges: what we know, what we want to know, and what we can know. *Chem. Commun.* **2022**, *58*, 327–342.

(44) Campbell, J.; Papp, T. WIDTHS OF THE ATOMIC K–N7 LEVELS. Atomic Data and Nuclear Data Tables **2001**, *77*, 1–56.

(45) Fuggle, J. C.; Alvarado, S. F. Core-level lifetimes as determined by x-ray photoelectron spectroscopy measurements. *Phys. Rev. A* **1980**, *22*, 1615–1624.

(46) Shannon, R. D. Revised effective ionic radii and systematic studies of interatomic distances in halides and chalcogenides. *Acta Crystallographica Section A* **1976**, *32*, 751–767.
